# Partial observability and management of ecological systems

**DOI:** 10.1002/ece3.9197

**Published:** 2022-09-13

**Authors:** Byron K. Williams, Eleanor D. Brown

**Affiliations:** ^1^ U.S. Geological Survey Science and Decisions Center Reston Virginia USA

**Keywords:** decision strategy, Markov decision process, partial observability, system dynamics, uncertainty

## Abstract

The actual state of ecological systems is rarely known with certainty, but management actions must often be taken regardless of imperfect measurement (partial observability). Because of the difficulties in accounting for partial observability, it is usually treated in an ad hoc fashion, or simply ignored altogether. Yet incorporating partial observability into decision processes lends a realism that has the potential to improve ecological outcomes significantly. We review frameworks for dealing with partial observability, focusing specifically on dynamic ecological systems with Markovian transitions, i.e., transitions among system states that are influenced by the current system state and management action over time. Fully observable states are represented in an observable Markov decision process (MDP), whereas obscure or hidden states are represented in a partially observable process (POMDP). POMDPs can be seen as a natural extension of observable MDPs. Management under partial observability generalizes the situation for complete observability, by recognizing uncertainty about the system's state and incorporating sequential observations associated with, but not the same as, the states themselves. Decisions that otherwise would depend on the actual state must be based instead on state probability distributions (“belief states”). Partial observability requires adaptation of the entire decision process, including the use of belief states and Bayesian updates, valuation that includes expectations over observations, and optimal strategy that identifies actions for belief states over a continuous belief space. We compare MDPs and POMDPs and highlight POMDP applications to some common ecological problems. We clarify the structure and operations, approaches for finding solutions, and analytic challenges of POMDPs for practicing ecologists. Both observable and partially observable MDPs can use an inductive approach to identify optimal strategies and values, with a considerable increase in mathematical complexity with POMDPs. Better understanding of POMDPs can help decision makers manage imperfectly measured ecological systems more effectively.

## Graphical abstract

The actual state of ecological systems is rarely known with certainty, but management actions must often be taken regardless of imperfect measurement (partial observability). We highlight applications of partially observable Markov decision processes (POMDPs) for some common ecological problems in order to clarify structure and operations, approaches for finding solutions, and analytic challenges of POMDPs for practicing ecologists. Incorporating partial observability into decision processes lends a realism that has the potential to improve ecological outcomes significantly.

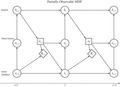

## 1 INTRODUCTION

Many important issues in ecology and management of ecological systems concern the behavior of dynamic systems in the presence of uncertainty. But changing ecological status and associated uncertainties can present real challenges to effective management (Nicol et al., 2015; Williams et al., 2002). For example, with dynamic systems smart decision making over an extended time must account for the potential effects of both current and future actions. A large body of theory and methodology has been developed over many decades to assess the optimal control of dynamic systems, although the size and complexity of problems to which it can be applied remain limited (Bertsekas, 2017).

Uncertainty about the actual state of an ecological system and its processes presents additional challenges. In ecology, a major source of uncertainty is partial observation (or imperfect measurement) of status over time. System dynamics are almost always tracked with sampling that leaves much of the system unobserved and subject to estimator imprecision (Williams & Brown, 2019). This is the case even with the most carefully designed and intensive sampling effort.

The challenges presented by imperfect observability are clearly seen in animal ecology and conservation. For example, the inadequacy of treating counts of animals as if they are known abundances has become widely recognized. That counts reflect the degree of detection during sampling is by now universally accepted, and much of the methods literature in recent years has dealt with sampling processes that account for partial detectability. In contrast, imperfect observability has been integrated into ecological management decision methods only to a very limited extent, largely because of the complexity of decision processes that incorporate estimated (imperfectly known) state and other variables, and the computational difficulties of implementing associated methods even with relatively small problems. There is a clear need to go beyond treating partial observability in sampling and estimation, by expanding its integration further into actual decision making.

We focus on ecological systems that are managed and tracked over time, and specifically on Markov decision processes, i.e., processes for which the probability of transition between successive states at any point depends only on the state and action taken at that time (Puterman, 1994). We use a standard objective for decision making of maximizing the accumulation of discounted returns over time.

The observability of the actual state of an ecological system when decisions are being made determines the type of Markov process. Markovian transitions among observable states are represented in an observable Markov decision process (MDP), whereas transitions among partially observable states are represented in a partially observable process (POMDP). Most aspects of a Markovian control problem must be adapted to partial observability, including transitions among states, valuation, and status tracking.

Many ecological problems lend themselves to a treatment with POMDPs. A common situation involves a partially observable resource that is subject to sequential decision making and monitoring over an extended time. To date, POMDPs have been applied to a limited number of ecological management and conservation problems for which accurate measurement is difficult or expensive. Among the most common of these are management of cryptic endangered species (Chadès et al., 2008; McDonald‐Madden et al., 2011; Tomberlin, 2010a); control of invasive plant species (Regan et al., 2011) and animal species (Kling et al., 2017; Peron et al., 2017; Rout et al., 2014), especially invasive forest pests (Fackler & Haight, 2014; Fackler & Pacifici, 2014; Haight & Polasky, 2010); and commercial fisheries (Lane, 1989; Memarzadeh et al., 2019; Memarzadeh & Boettiger, 2018). These and other examples are highlighted in Table 1.

**TABLE 1:** Applications of partially observable Markov decision processes in ecology

Author(s)	Resource Context	Species	Actions	Features	Solution approach
Chadès et al. (2008)	Endangered species	Sumatran tiger *Panthera tigris sumatrae*	Manage habitat, survey, do nothing	2 states (extant/extinct), 3 actions, 3 time steps	Analytical solution, incremental pruning
Fackler and Pacifici (2014)	Pest infestation	General pest species	Unspecified	2 hidden models, environment signals used for updating model uncertainty	State space discretization, stochastic dynamic programming
Haight and Polasky (2010)	Invasive species	Forest pest	Monitoring only, treatment only, both, or neither	Discretized approximation of belief space for 3 states	Customized FORTRAN program for 4 actions over 20 time periods
Lane (1989)	Commercial fishing	Salmon (*Oncorhynchus* spp.)	Intra‐seasonal fishing within geographic fishing zones	Finite states for each zone; actions are fishing within a zone, or not	Sondik one‐pass algorithm
McDonald‐Madden et al. (2011)	Endangered species	Sumatran tiger	Survey, management, do nothing	Considers 2 populations, compares results with perfect observability	Incremental pruning
Memarzadeh and Boettiger (2018)	Marine fisheries	Argentine hake *Merluccius hubbsi*	Fish harvest	Handles either or both structural uncertainty and partial observability	Point‐based value iteration with SARSOP
Regan et al. (2011)	Invasive species	Branched broomrape *Orobanche ramosa*	Low cost and inefficient action, high cost but efficient action, do nothing	3 states, 3 actions, 2 observations	Stochastic dynamic programming
Rout et al. (2014)	Invasive species	Black rat *Rattus rattus*	Quarantine, control, mixture	3 states, 3 actions	Incremental pruning
Tomberlin (2010b)	Endangered seabird habitat	Marbled murrelet *Brachyramphus marmoratus*	Monitoring, habitat management	2 occupancy states, 2 actions, 5 time steps	Eagle/Monahan algorithms
Pascal et al. (2020)	Threatened and endangered species	Sumatran tiger	Manage, monitor	Framed in terms of the termination of management or surveying, 2 observations	Point‐based value iteration with SARSOP
Tomberlin (2010a)	Erosion control on roads in redwood forests	Forestry	High or low monitoring effort, erosion treatment, no action	High‐ and low‐level erosion condition	Backward recursion with α−vectors over finite time
Nicol et al. (2015)	Management of shorebird habitats under changing sea‐level conditions	10 species of migratory shorebirds	Protection of non‐breeding sites against sea‐level change	Factored MOMDP with observed states, uncertain and non‐stationary transition structure	Point based value iteration with Symbolic Perseus
Nicol and Chadès (2012)	Marine oil spill risks	Sea otter *Enhydra lutris*	Reduction or cleanup of oil spills, reintroduction, monitoring	Discretization of a continuous state with CU‐tree^a^	Point‐based value iteration with Perseus
Kling et al. (2017)	Marine species in a preserve	Lionfish *Pterois* spp.	Lionfish removal, monitoring	Observations based on removal effort and presence/absence of monitoring	Projected belief MDP
Memarzadeh et al. (2019)	Commercial fisheries	Multiple fish species	Fishing catch quotas	Compares MSY, MDP, and POMDP solutions	Point‐based value iteration with SARSOP
Sloggy et al. (2020)	Commercial forestry	Loblolly pine *Pinus taeda*	Monitoring, harvest, regeneration, delay	Valuation based on stochastic price and harvest amounts	Projected belief MDP
Fackler and Haight (2014)	Invasive species	Forest pest	Treatment only, monitoring, both monitoring and treatment, no action	Discretization of a continuous state	Monahan's exact method, variations on Lovejoy's discretization method
Fackler et al. (2014)	Recreation management	Golden eagle *Aquila chrysaetos*	Management of recreation near nesting sites	Observable occupancy, uncertain disturbance effect	Unspecified
MacLachlan et al. (2017)	Bovine tuberculosis in cattle herds	Domestic cattle *Bos taurus*	Herd testing for infection, followed by isolation if found	3 states for a herd, testing and control of multiple herds	Projected belief MDP
Sethi et al. (2005)	Commercial fisheries	Commercial fish species	Fish harvest quotas	Discrete observations, continuous states and actions; spline interpolations	Value function iteration for infinite time horizon
Chadès et al. (2021)	Australian birds threatened by habitat degradation and predation	Gouldian finch *Erythrura gouldiae*	Fire and grazing management, feral cat control, provide nesting boxes, do nothing	Observable states, stationary but unknown state transitions	MOMDP (“hidden model MDP”) with discrete states and models, several solution algorithms

^a^ See Uthe and Veloso (1998).

Importantly, incorporating partial observability into decision processes lends a realism that has the potential to improve ecological outcomes. For example, McDonald‐Madden et al. (2011) showed that accounting for partial observability led to better strategic outcomes in conservation planning to save the last remaining wild Sumatran tigers (*Panthera tigris sumatrae*). Realism can be especially important in a regulatory context such as commercial fisheries, where standard models that assume perfect measurements of a stock can lead to harvest decision rules that cause fishery collapse, as in the case of the Argentine hake *Merluccius hubbsi* (Memarzadeh & Boettiger, 2018). In contrast, Memarzadeh et al. (2019) demonstrated that POMDP‐based decision methods could avoid unintentional extinctions, and lead to consistently higher rates of recovery of depleted fish stocks.

In this paper, we compare completely and partially observed Markov decision processes for dynamic ecological systems that are managed and tracked over time. A comparison of MDPs and POMDPs highlights analytic and operational similarities between these two situations and clarifies the increased complexity one confronts when realistically accounting for limited observability. We build on recent ecological literature (e.g., Chadès et al., 2021; Williams, 2009, 2011) and provide additional detail for ecologists who wish to understand the mechanics of POMDPs. We describe specifications, policies, valuations, and solution approaches for observable and partially observable MDPs. In addition, we discuss model extensions, infinite versus finite time horizons, mixed observability processes, adaptive management with POMDPs, nonstationary models, and continuous states in considerable detail.

**FIGURE 1:**
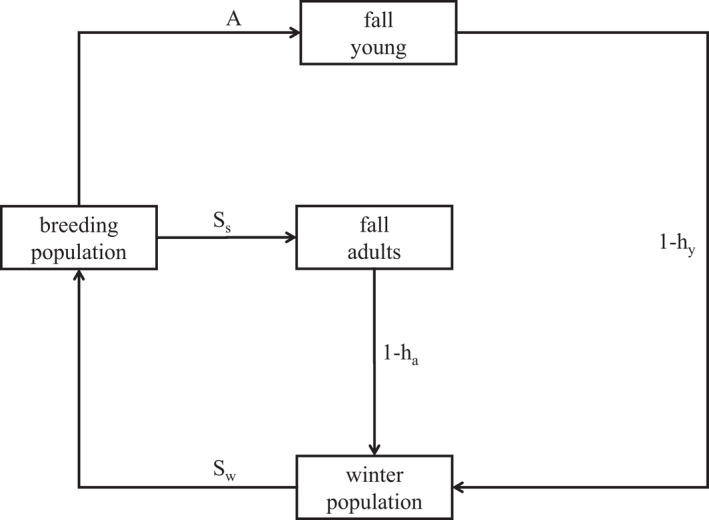
Model of waterfowl population dynamics. Includes survival rates for spring–summer $\left(S_{s}\right)$ and fall–winter $\left(S_{w}\right)$, along with harvest rates for young $\left(a_{y}\right)$ and adults $\left(a_{a}\right)$ and age ratio $\left(A\right)$ for reproduction/recruitment.

In the following sections, we illustrate the concepts of POMDPs with examples from long‐term sport hunting of waterfowl in North America. Waterfowl hunting has been regulated for over a century by U.S. federal law and international agreement, and managed since 1995 through the annual setting of hunting regulations under the rubric of “adaptive harvest management” (Johnson et al., 2015; Williams & Johnson, 1995). Harvest management relies on simple models of waterfowl population dynamics that are based on hypotheses about the impact of harvest on annual survivorship and the importance of density dependence in recruitment (Figure 1). Models incorporating different hypotheses produce different population trajectories, and model effectiveness can be evaluated by comparing these trajectories against observations from annual population monitoring. Such a framework can be used to investigate optimal harvest strategies in the presence of partial observability, as well as imperfect understanding of population dynamics (Williams, 2011).

## 2 PROCESS SPECIFICATION

In this section, we introduce the general elements of Markov decision processes, including system states, transitions among states, observations, management actions, returns (or rewards), discount factors, and time horizons. These elements provide a foundation for describing dynamic ecological systems that are managed over time.

In an ecological context, decision making over time builds on transitions among states, as influenced by management actions in concert with ecological processes such as mortality, reproduction, and movement. Stochastic variation in the transitions can be described with transition probabilities in a stochastic process, or in the case of Markovian transitions, a Markov decision process. In our example of waterfowl harvests, the change in population size from 1 year to the next is held to be influenced by the current population size, environmental conditions and the amount of harvest in the fall. Stochasticity in population size the ensuing year is a result of environmental fluctuations, randomness in the influence of hunting regulations, and stochastic biological processes that produce change.

A formal specification of a Markov decision process, whether partially or completely observable, must account for system dynamics and management returns over some time horizon. More specifically, it includes the duration of the process, a characterization of system state, probabilities of transition among states at each time step, and a value function that aggregates returns to management over time. Ecological status is assumed to be tracked as decisions are made at discrete times. We assume initially that there are finitely many possible states and actions at each point over a finite time horizon, and later consider continuous‐state and infinite‐time POMDPs.

### 2.1 Specification of observable MDPs

A controlled process with Markovian transitions among observable states is characterized as follows.

*Notation*:

x, state of an ecological system, which for an MDP is observable.

*a*, action that influences returns and transitions among states (“state transitions”)

*State transitions*:

$$x^{\prime }=F\left(x,a,z\right)$$

with random environmental conditions z, from which are produced probabilities of transition

$$P\left(x^{\prime }|x,a\right)$$

from state *x* to state $x^{\prime }$, given that action *a* is taken.

*Returns*:

Immediate returns $R\left(a|x\right)$ are assumed to depend on the system's state and the action taken in that state. If returns are based on transitions, then $R\left(a|x\right)=\sum_{x^{\prime }}P\left(x^{\prime }|x,a\right)R\left(a|x,x^{\prime }\right)$.

*MDP specification*:

An observable MDP is specified by the tuple $\left\{X,A,P,R,T,\lambda \right\}$, where

- *X* is the set of system states *x*. Examples could include population size or density, population vital rate, spatial distribution, biodiversity, and habitat features.
- *A* is the set of actions *a* that are available to a manager, potentially including monitoring as well as conservation actions. Examples could include selection of hunting limits, introduction or removal of species, habitat manipulation, contaminant clean‐up, adaptations to climate change, regulatory actions, and field sampling designs.
- *P* is a transition probability function specifying probabilities $P\left(x^{\prime }|x,a\right)$ of transition from state *x* to state $x^{\prime }$, given that action *a* is taken. The conditional probability $P\left(x|x,a\right)$ corresponds to no change, and $\sum_{x^{\prime }}P\left(x^{\prime }|x,a\right)=1$.
- *R* is a return or reward function, with $R\left(a|x\right)$ the immediate return when action *a* is taken and the system is in state *x*. For example, returns could be measured in terms of population survival rate, number of animals, increase in biodiversity, risk abatement, economic profit, and opportunity cost.
- *T* is the terminal time of a time horizon consisting of equal time steps between an initial time and *T*, which could be infinite.
- λ is a discount factor between 0 and 1 that relates future returns to present value. As λ declines from unity, future returns become less important relative to immediate returns.

**FIGURE 2:**
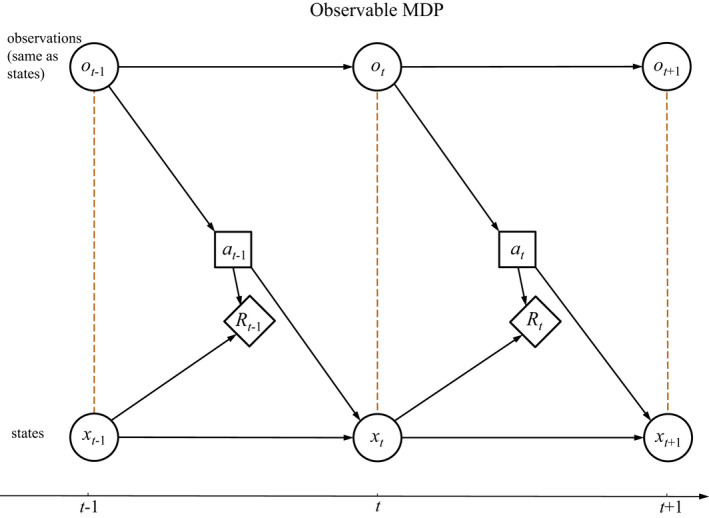
Influence diagram for an observable Markov decision process

In an observable MDP, observations coincide with actual states. At any time, the state affects the selection of an action and influences returns and transitions to subsequent states (Figure 2). Actions in turn influence state transitions and returns.

The observable MDP framework can be applied to our example of the sport harvest of waterfowl. Thus, state *x* represents population size at a given point in time, $x^{\prime }$ is the population size at the next time, *a* is the harvest rate targeted by current regulations, *z* represents environmental conditions (e.g., spring precipitation), and $R\left(a|x\right)$ is the amount of harvest for harvest rate *a*, given the population size *x*. The state transition function $x^{\prime }=F\left(x,a,z\right)$ describing population change from one time to the next is held to be understood and well specified, and the population size is assumed to be known with certainty (or can effectively be treated as such) at each point in time.

### 2.2 Specification of partially observable MDPs

POMDPs extend the framework of observable MDPs by including observations that differ from, but are related to, the unobservable system states. A common situation is for the observations to produce estimates of the system state (Nichols & Williams, 2006), although in general the only requirement is a statistical association between observations and the process state. Like the observable states in an MDP, observations in a POMDP are used to track changes in status over time. A Markov decision process with transitions among unobservable states is characterized by the following additional features and adaptations.

*Notation*:

x, system state, which is unobservable

*a*, action that influences returns, state transitions, and (possibly) observations

o, observation (usually assumed to be discrete) that is associated with, but not the same as, system state

b, belief state, with $b\left(x\right)$ the probability associated with state x

*Observation function*:

$$o^{\prime }=G\left(x^{\prime },a,\varepsilon \right)$$

producing random observations $o^{\prime }$, with probabilities

$$f\left(o^{\prime }|x^{\prime },a\right).$$

Actions may or may not influence observations; if not, the observation probabilities reduce to $f\left(o^{\prime }|x^{\prime }\right)$. Initially, we assume observation $o^{\prime }$ is tied to the posterior system state $x^{\prime }$ after implementation of prior action *a*. Later, we consider a different order for observations and state updates. In some but not all cases, observations can be expressed as data‐based estimators.

*Returns*:

Immediate returns are averaged over belief state *b*:

$$R\left(a|b\right)=\sum_{x}b\left(x\right)R\left(a|x\right).$$

*POMDP specification*:

A POMDP generalizes $\left\{X,A,P,R,T,\lambda \right\}$ for observable MDPs, by allowing states to be only partially observable and appending a probability distribution for observations in an observation space *O*. Thus, a POMDP is specified by the tuple $\left\{X,A,O,P,f,R,T,\lambda \right\}$, where

- *O* is a set of potential observations *o*, obtainable through activities such as field sampling, modeling, or laboratory assessments.
- *f* is an observation function, with $f\left(o^{\prime }|x^{\prime },a\right)$ the probability that $o^{\prime }$ is observed, given state $x^{\prime }$ and action *a*.

**FIGURE 3:**
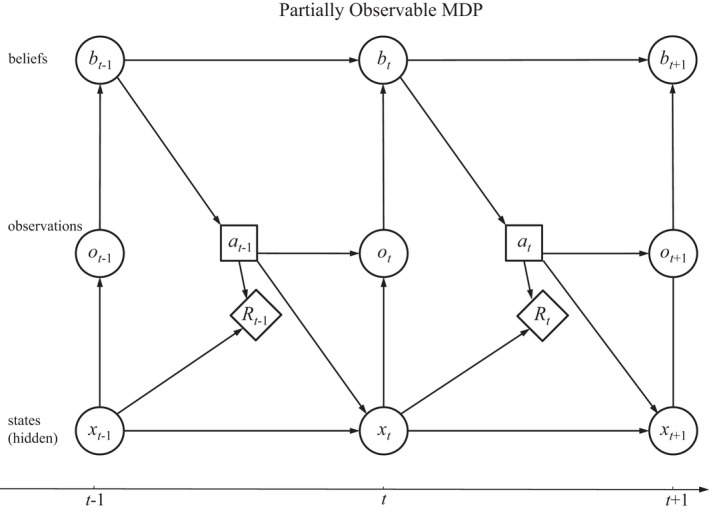
Influence diagram for a partially observable Markov decision process (after Chadès et al., 2021).

Because the states are themselves unobservable, ecological status must be tracked with belief states. At any time the actual state of the system influences immediate returns, transitions to subsequent states, and observations, but not actions (Figure 3). Observations are used to update belief states, which in turn inform the selection of actions. Finally, actions control transitions, returns, and (possibly) observations. A comparison of Figures 2 and 3 makes it clear that the framework for POMDPs extends that of an observable MDP, by incorporating observations that differ from the actual system states and introducing belief states to track the system's status over time.

In our waterfowl example, the only difference in the frameworks for partial and complete observability concerns the observability of population size *x*. For the POMDP framework, *x* cannot be observed directly and must be tracked with data *o* that are obtained through field sampling. The data are combined into an estimator of population size that is associated with the actual population size, with randomness inherited from sampling and estimation protocols. For this situation, the estimator distribution serves as the population belief state.

The use of belief states to track the status of the system is a critical feature distinguishing POMDPs from observable MDPs. The states in an observable MDP typically are discrete and countable, and define a finite state space. Given finitely many actions, it is theoretically possible to list all state/action combinations and compare them in evaluating MDP policies. For a POMDP with finitely many actions and observations, it also is possible to identify all action/observation combinations for a particular belief state. However, any effort to do so over all action/belief state combinations is defeated by the continuous nature of a belief space comprising infinitely many belief states. As discussed later, a different approach from that for MDPs must be taken to evaluate a POMDP, i.e., one that explicitly accounts for a continuous belief space.

## 3 PROCESS POLICY

In this section we describe policies for a Markov decision process in terms of time‐specific states, observations, and actions, and characterize policies for both observable and partially observable MDPs in terms of *policy trees*. The notation for policy trees highlights the linkages between observable MDPs and POMDPs.

The trajectory of a Markov decision process over its time horizon is controlled by the temporal sequence of decisions imposed on the process, i.e., the process policy. A policy extends the notion of a time‐specific action influencing system transitions, to include actions and transitions over the duration of the process. Thus, it identifies actions that are tied to the status of the system at every point in the time horizon. The sequence of state‐based decisions for a Markov process is a defining part of the process, in that state trajectories, values, patterns of actions, and recurrences among states are all influenced by the process policy.

For observable MDPs, a policy essentially assigns an action *a* for every system state x∈X at every time over the duration of the process. On the other hand, a policy for partially observable MDPs assigns an action *a* for every belief state *b* at every point in time. Policies for both MDPs and POMDPs can be described with actions that are hierarchically organized in policy trees (Kaelbling et al., 1998).

### 3.1 Policy for observable MDPs

**FIGURE 4:**
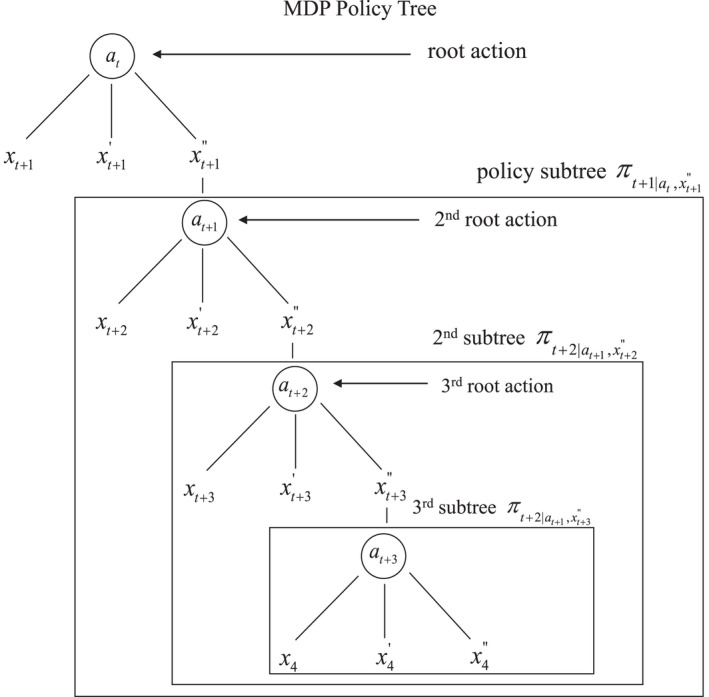
Policy tree for an observable Markov decision process

A policy tree for an observable Markov decision process displays actions and (observable) states over the course of the process time horizon $\left\{t,\ldots ,T\right\}$. A tree is arranged temporally, with a root action followed in sequence by states and actions at later times (Figure 4). If action $a_{t}$ is taken at *t*, the sub‐tree $\pi_{t+1|a_{t},x^{\prime }}$ consists of actions for states over the remainder of the time horizon. By construction, policy tree $\pi_{t}$ is simply a root action $a_{t}$ and sub‐trees for all subsequent states $x^{\prime }$, that is,

$$\pi_{t}=\left\{a_{t},\pi_{t+1|a_{t},x^{\prime }}|x^{\prime }\in X\right\}.$$

Because of the hierarchical nature of a policy tree, any state at any time could be thought of as a starting point, with the action for that state considered to be the root action of a policy tree. This allows one to essentially “decompose” a policy into a temporal hierarchy, in which the decision‐making framework at a given time subsumes all decisions for later times, and is itself subsumed in the decision‐making frameworks for earlier times. As discussed in the next section, this hierarchical clustering allows a concise representation of iterative valuation and policy determination.

In our waterfowl example, a policy tree under full observability simply consists of hunting regulations each year for each population size. A particular trajectory of population sizes over time will have an associated sequence of hunting regulations, which fluctuate over time as the population does. And at any particular time, the range of regulations for a policy will be tied to the possible population sizes at that time. Regulatory variation across sizes and times is expressed in the notation $\pi_{t}=\left\{a_{t},\pi_{t+1|a_{t},x^{\prime }}|x^{\prime }\in X\right\}$.

### 3.2 Policy for partially observable MDPs

**FIGURE 5:**
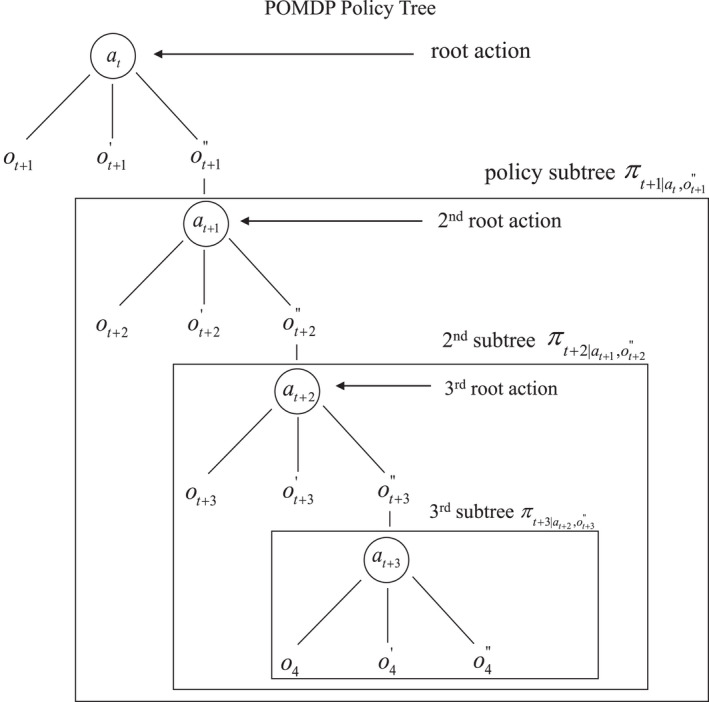
Policy tree for a partially observable Markov decision process

Because system states are not observed under partial observability, policy trees for a POMDP must be based on observations rather than the (unobservable) states themselves. A POMDP policy tree has a root action followed in sequence by observations and actions at later times (Figure 5). If action $a_{t}$ is taken at *t*, the sub‐trees $\pi_{t+1|a_{t},o^{\prime }}$ consist of actions for later observations over the duration of the process. By construction, policy tree $\pi_{t}$ is simply the combination of a root action $a_{t}$ and sub‐trees for all possible observations $o^{\prime }$, that is,

$$\pi_{t}=\left\{a_{t},\pi_{t+1|a_{t},o^{\prime }}|o^{\prime }\in O\right\}.$$

As with observable MDPs, the clustering of policy trees for POMDPs allows iterative valuation and policy determination to be concisely represented.

In our waterfowl hunting example, a policy tree under partial observability consists of hunting regulations each year for each estimate of population size based on the field data. A particular trajectory of data‐based estimates over time will have an associated sequence of hunting regulations. And at any particular time, the range of regulations will be tied to the possible population estimates at that time. Regulatory variation across data and times is expressed by the notation $\pi_{t}=\left\{a_{t},\pi_{t+1|a_{t},o^{\prime }}|o^{\prime }\in O\right\}$.

## 4 PROCESS VALUATION

In this section, we discuss valuation for observable MDPs and POMDPs, including optimal valuation. We clarify how valuation is actually determined with step‐by‐step procedures for finding policy‐based values, and we describe some procedural alternatives found in the literature for optimal policy and valuation.

The value function serves as a metric for comparing as well as measuring performance of policies for a decision process. For observable MDPs, it aggregates returns for an MDP policy tree, starting in state *x* at time *t*. For partially observable MDPs, it aggregates returns for a POMDP policy tree starting in belief state *b* at time *t*. In both cases, the value function can be used to compare policies and identify an optimal policy.

### 4.1 Valuation with observable MDPs

Valuation for completely observable Markov decision processes can be described in terms of policy trees $\pi_{t}=\left\{a_{t}|\pi_{t+1|a_{t},x^{\prime }},x^{\prime }\in X\right\}$, each tree having an associated vector of state‐specific components

(1) $$V_{\pi_{t}}\left(x\right)=R\left(a_{t}|x\right)+\lambda \sum_{x^{\prime }}P\left(x^{\prime }|x,a_{t}\right)V_{\pi_{t+1|a_{t},x^{\prime }}}\left(x^{\prime }\right)$$

(see Appendix S1). The value function in Equation (1) includes an immediate return $R\left(a_{t}|x\right)$ along with future values $V_{\pi_{t+1\|a_{t},x^{\prime }}}\left(x^{\prime }\right)$ that are averaged over the system states $x^{\prime }$. Calculation of $V_{\pi_{t}}\left(x\right)$ thus involves two steps:

- averaging the posterior values $V_{\pi_{t+1|a_{t},x^{\prime }}}\left(x^{\prime }\right)$ with transition probabilities $P\left(x^{\prime }|x,a_{t}\right)$; and
- discounting the average posterior value with λ and adding the immediate return $R\left(a_{t}|x\right)$ to get $V_{\pi_{t}}\left(x\right)$.

A more concise expression for the value function is

(2) $$V_{\pi_{t}}\left(x\right)=R\left(a_{t}|x\right)+\lambda V_{\pi_{t+1}}^{\prime }\left(x|a_{t}\right),$$

where $V_{\pi_{t+1}}^{\prime }\left(x|a_{t}\right)$ represents a transformation of future values in Equation (1) by the transition probabilities, i.e.,

$$V_{\pi_{t+1}}^{\prime }\left(x|a_{t}\right)=\sum_{x^{\prime }}P\left(x^{\prime }|x,a_{t}\right)V_{\pi_{t+1|a_{t},x^{\prime }}}\left(x^{\prime }\right).$$

The assessment of a decision process typically involves a search for policies that can produce the highest value. To obtain optimal valuation with observable MDPs, the values and policies in Equation (1) can be optimized at each time with the Bellman equation (Bellman, 1957), by means of backward recursion (Bertsekas, 2012). From Equation (1), optimal valuation can be expressed as

$$V_{t}\left[x\right]=\max_{a}\left\{R\left(a|x\right)+\lambda \sum_{x^{\prime }}P\left(x^{\prime }|x,a\right)\;\max_{\pi_{t+1}}\;V_{\pi_{t+1}}\left(x^{\prime }\right)\right\}$$

(see Appendix S1). Thus, the optimal value for a state *x* is produced in a two‐step procedure:

- optimize future returns $V_{\pi_{t+1}}\left(x^{\prime }\right)$ over the possible trees at *t* + 1; and
- optimize the sum $R\left(a|x\right)+\lambda \sum_{x^{\prime }}P\left(x^{\prime }|x,a\right)\;V_{\pi_{t+1}^{*}}\left(x^{\prime }\right)$ over *a*

(see Williams et al., 2002; Marescot et al., 2013 for details). Optimal valuation can also be expressed in terms of Equation (2) by

(3) $$V\left[x\right]=\max_{a_{t}}\left\{R\left(a_{t}|x\right)+\lambda \;\max_{\pi_{t+1}}\;V_{\pi_{t+1}}^{\prime }\left(x|a_{t}\right)\right\}.$$

In our waterfowl example, with observable population status, the value function for a population of size $x_{0}$ starting at time *t* = 0 can be represented simply as the expected sum of current and future harvest amounts over the problem time horizon, $V\left(x_{0}\right)=E\left[R\left(a_{0}|x_{0}\right)+\sum_{t=1}^{T}\lambda^{t}R\left(a_{t}|x_{t}\right)\right]$, where future population states are described in terms of Markov transitions as above. We note that such a value function is intrinsically conservation oriented, in that current harvest, by influencing the status of future populations, must account for future harvest yields.

### 4.2 Valuation with partially observable MDPs

Valuation for partially observable Markov decision processes is based on policy trees $\pi_{t}=\left\{a_{t},\pi_{t+1|a_{t},o^{\prime }}|\;o^{\prime }\in O\right\}$. Every tree $\pi_{t}$ has associated with it a vector of state‐specific values

(4) $$V_{\pi_{t}}\left(x\right)=R\left(a_{t}|x\right)+\lambda \sum_{x^{\prime }}P\left(x^{\prime }|x,a_{t}\right)\sum_{o^{\prime }}f\left(o^{\prime }|x^{\prime },a_{t}\right)\;V_{\pi_{t+1}|a_{t},o^{\prime }}\left(x^{\prime }\right)$$

(see Appendix S1). The value function in Equation (4) includes an immediate return $R\left(a_{t}|x\right)$ for a prior state *x*, along with future values $V_{\pi_{t+1}|a,o^{\prime }}\left(x^{\prime }\right)$ averaged over observations $o^{\prime }$ as well as posterior states $x^{\prime }$. A comparison of Equations (1 and 4) shows that valuation of a POMDP has the same general form as that of an MDP, except $V_{\pi_{t+1|a_{t},x^{\prime }}}\left(x^{\prime }\right)$ in Equation (1) is replaced by the average value

$$\sum_{o^{\prime }}f\left(o^{\prime }|x^{\prime },a\right)\;V_{\pi_{t+1|a_{t},o^{\prime }}}\left(x^{\prime }\right)$$

in Equation (4).

Because the state *x* of a partially observable process is not known, actual valuation must be based on a belief state *b*, with $V_{\pi_{t}}\left(x\right)$ averaged over *b*:

$$V_{\pi_{t}}\left(b\right)=\sum_{x}b\left(x\right)V_{\pi_{t}}\left(x\right).$$

In the Appendix S1, we describe two useful forms for computing $V_{\pi_{t}}\left(b\right)$. One uses a transformation of future values with the transition probabilities

$$V_{\pi_{t+1}}^{\prime }\left(x|a_{t},o^{\prime }\right)=\sum_{x^{\prime }}P\left(x^{\prime },o^{\prime }|x,a_{t}\right)V_{\pi_{t+1|a_{t},o^{\prime }}}\left(x^{\prime }\right)$$

to express valuation as

(5) $$\begin{array}{c} \begin{array}{c} V_{\pi_{t}}\left(b\right)=R\left(a_{t}|b\right)+\lambda \sum_{o^{\prime }}\sum_{x}b\left(x\right)V_{\pi_{t+1}}^{\prime }\left(xa_{t}|o^{\prime }\right),\\ \;=R\left(a_{t}|b\right)+\lambda \sum_{o^{\prime }}V_{\pi_{t+1}}^{\prime }\left(b|a_{t},o^{\prime }\right). \end{array} \end{array}$$

Note that Equation (5) has the same general form as Equation (2) for observable MDPs, except $V_{\pi_{t+1}}^{\prime }\left(x|a\right)$ in Equation (2) is replaced by the aggregated value

$$\sum_{o^{\prime }}V_{\pi_{t+1}}^{\prime }\left(b|a_{t},o^{\prime }\right)$$

in Equation (5). The effect of partial observability is thus to require an aggregation of values over the observations.

An alternative but equivalent form for $V_{\pi_{t}}\left(b\right)$ uses Bayesian updating of beliefs,

$$\begin{array}{c} b_{a,o^{\prime }}\left(x^{\prime }\right)=\frac{P\left(o^{\prime },x^{\prime }|b,a\right)}{P\left(o^{\prime }|b,a\right)}\;\\ =\frac{\sum_{x}P\left(o^{\prime },x^{\prime }|x,a\right)b\left(x\right)}{P\left(o^{\prime }|b,a\right)}, \end{array}$$

to get

(6) $$V_{\pi_{t}}\left(b\right)=R\left(a|b\right)+\lambda \sum_{o^{\prime }}P\left(o^{\prime }|b,a\right)V_{\pi_{t+1}|a,o^{\prime }}\left(b_{a,o^{\prime }}\right).$$

The forms in Equations (5 and 6) produce the same values for all belief states in the belief space.

Value expressions (5 and 6) both can be used to compute optimal values for a POMDP. Optimal values based on Equation (5) are given by

(7) $$V_{t}\left[b\right]=\max_{a_{t}}\left\{R\left(a_{t}|b\right)+\lambda \sum_{o^{\prime }}\max_{\pi_{t+1}}\;V_{\pi_{t+1}}^{\prime }\left(b|a_{t},o^{\prime }\right)\right\},$$

and optimal values based on Equation (6) are given by

(8) $$\begin{array}{c} \begin{array}{c} V_{t}\left[b\right]=\max_{\pi_{t}}\sum_{x}b\left(x\right)V_{\pi_{t}}\left(x\right)\;\\ =\max_{a_{t}}\left\{R\left(a_{t}|b\right)+\lambda \sum_{o^{\prime }}P\left(o^{\prime }|b,a_{t}\right)\max_{\pi_{t+1}}V_{\pi_{t+1}}\left(b_{a_{t},o^{\prime }}\right)\right\} \end{array} \end{array}$$

(see Appendix S1).

As with observable MDPs, expressions (7 and 8) both involve two optimizations, one over trees for time *t* + 1 and one over actions at time *t*. A comparison of Equations (3 and 7) shows that MDPs and POMDPs have analogous formats for optimization, except the latter equation includes an aggregation of optimal future values across observations.

In our waterfowl example, with harvest regulations based on partially observable populations, the value function for a population with belief state $b_{0}$ starting at time *t* = 0 can be represented simply as the expected sum of current and future harvest amounts over the problem time horizon, $V\left(b_{0}\right)=E\left[R\left(a_{0}|b_{0}\right)+\sum_{t=1}^{T}\lambda^{t}R\left(a_{t}|b_{t}\right)\right]$. In this case, future belief states are tied to observations through Bayes' theorem, as above. As with complete observability, accounting for future harvests means that the current harvest, by influencing future population status, must account for future harvest yields.

### 4.3 Standard versus extended models

In the standard POMDP model for state transitions, observations are held to occur after state transitions, without directly affecting the state transition probabilities. An alternative model allows observations to occur before state transitions. By incorporating a different sequencing of observations and state transitions, an alternate or extended model allows one to consider many problems not easily accommodated by the standard model, namely those in which observations can influence the transition probabilities. In our waterfowl hunting example, observations of waterfowl harvest in the fall can produce updated beliefs before winter mortality and spring reproduction affect next year's population state, and thus can influence the transitions used in the valuation of harvest strategies.

The operational difference between the standard and extended models is seen by a comparison of belief‐updating and the respective value functions. With the standard model, observations occur after the state transitions,

$$x,a\to x^{\prime }\to o^{\prime },$$

so that observations $o^{\prime }$ do not influence the transition probabilities $P\left(x^{\prime }|x,a\right)$. Belief states are updated by

$$\begin{array}{c} b_{a,o^{\prime }}\left(x^{\prime }\right)=\frac{P\left(x^{\prime },o^{\prime }|b,a\right)}{P\left(o^{\prime }|b,a\right)}\;\\ =\frac{f\left(o^{\prime }|x^{\prime },a\right)P\left(x^{\prime }|b,a\right)}{P\left(o^{\prime }|b,a\right)}, \end{array}$$

and the process value function averages immediate and future value over observations $o^{\prime }$:

$$V_{\pi_{t}}\left(b\right)=R\left(a|b\right)+\lambda \sum_{o^{\prime }}P\left(o^{\prime }|b,a\right)\;V_{\pi_{t+1|a,o^{\prime }}}\left(b_{a,o^{\prime }}\right)$$

(see Appendix S1).

On the other hand, with the extended model the observations occur before the state transitions,

$$x,a\to o\to x^{\prime },$$

so that observations o can influence the transition probabilities $P\left(x^{\prime }|x,a,o\right)$. Belief states are updated by

$$\begin{array}{c} b_{a,o}\left(x^{\prime }\right)=\frac{P\left(x^{\prime },o|b,a\right)}{f\left(o|b,a\right)}\;\\ =\frac{f\left(o|x,a\right)P\left(x^{\prime }|b,a,o\right)}{f\left(o|b,a\right)}, \end{array}$$

and the process value function averages immediate and future value over observations o:

(9) $$V_{\pi_{t}}\left(b\right)=R\left(a|x\right)+\lambda \sum_{o}f\left(o|b,a\right)\;V_{\pi_{t+1|a,o}}\left(b_{a,o}\right)$$

(see Appendix S1). The value function shown in Equation (9) for the extended model differs from that for the standard model only in the use of prior and posterior observations in the updating of beliefs and weighting of future values.

The extended model allows for assessment of many ecological problems that otherwise would be difficult or impossible to assess with the standard model. Fackler and Pacifici (2014) describe three examples representing different levels of dependence between observations and future states. One involves the observed harvest of an unobserved population, where the future population state is directly influenced by the observation of harvest in the prior year. Another example involves a treatment to reduce an unobserved pest infestation, where observed environmental conditions in the previous year influence future infestation. A third involves the control of avian nest predation, where observed predator numbers in the previous year influence predation and thus the future status of an avian population. Assessment in these and other cases is facilitated by the extended model, in which observations informing and possibly influencing management actions that affect future ecological conditions occur before the ecological transitions themselves.

## 5 SOLUTION APPROACHES

In this section, we consider the mechanics of different approaches to finding policies with optimal value. We discuss valuation by means of value iteration for both observable and partially observable MDPs. We describe the construct of α – vectors for POMDPs, and outline iterative approaches to optimal policy and valuation that use *α* vectors.

A key challenge in managing dynamic systems involves the number of decisions that can potentially be made over time. The number of possible policy trees for an observable MDP increases exponentially with an increasing number of states, actions, and length of the time horizon. Even more troubling for POMDPs is that a listing and evaluation of trees is not possible because of the continuous belief space. In fact, finite‐horizon POMDPs are PSPACE‐complete (Papadimitriou & Tsitsiklis, 1987), and infinite‐horizon POMDPs are undecidable (Madani et al., 2003). Thus, approximations of optimal solutions must be used for most problems.

### 5.1 Solution approaches with observable MDPs

The solution of an observable MDP yields optimal values $V_{t}\left[x\right]$ across a discrete state space at each time *t*. With finitely many states and actions, values for every policy tree could at least conceivably be listed for all states at each time, and optimal actions and values could be identified. However, such an exhaustive enumeration is prohibitively costly in terms of computing resources for all but small problems.

Finding optimal values and policies is greatly facilitated by value iteration, in which optimal valuation begins at the terminal time and proceeds backward to find optimal values that build on those previously identified (Marescot et al., 2013). Value iteration involves the following steps:

- determine the optimal value $V_{T}\left[x\right]=\max_{a}R\left(a|x\right)$ and optimal action $a_{T}^{*}=\arg\;\max_{a}\;R\left(a|x\right)$ for each state *x* at time *T*;
- determine optimal values $V_{T-1}\left[x\right]=\max_{a}\left\{R\left(a|x\right)\right.$ $\left.+\lambda \sum_{x^{\prime }}P\left(x^{\prime }|x,a\right)\;V_{T}\left[x^{\prime }\right]\right\}$ and optimal actions $a_{T-1}^{*}\left(x\right)=\arg\underset{a}{\;\max}\left\{R\left(a|x\right)+\lambda \sum_{x^{\prime }}P\left(x^{\prime }|x,a\right)\;V_{T}\left[x^{\prime }\right]\right\}$ for each state at time *T*–1; and
- determine $V_{t}\left[x\right]=\max_{a}\left\{R\left(a|x\right)+\lambda \sum_{x^{\prime }}P\left(x^{\prime }|x,a\right)\;V_{t+1}\left[x^{\prime }\right]\right\}$ in reverse sequence for each time t=0,1,…,T−2.

The final result is a policy that identifies optimal actions and values for all states over the time horizon. This approach, known as value iteration or dynamic programming, helps to alleviate the “curse of dimensionality” that otherwise can defeat attempts to find a solution (Bellman, 1957).

Dynamic programming has been used for a wide range of ecological problems (see, e.g., Marescot et al., 2013; Williams et al., 2002). In most cases, an ecological system is described in terms of Markovian transitions among finitely many observable states, and management actions that influence the transitions over an extended, often indefinite, time horizon. Objectives often optimize combinations of ecological production costs, management costs, and metrics of system status.

### 5.2 Solution approaches with partially observable MDPs

The solution of a POMDP consists of the optimal values $V_{t}\left[b\right]$ across a continuous belief space at each time *t*. With finitely many system states, actions, and observations, all combinations of these factors could be listed for any belief state. However, it is not possible to do so for all the infinitely many belief states in the continuous belief space of a POMDP, and thus not possible to enumerate values over the continuous space. This contrasts with the situation for observable MDPs over a space of finitely many states and requires a substantially different method.

**FIGURE 6:**
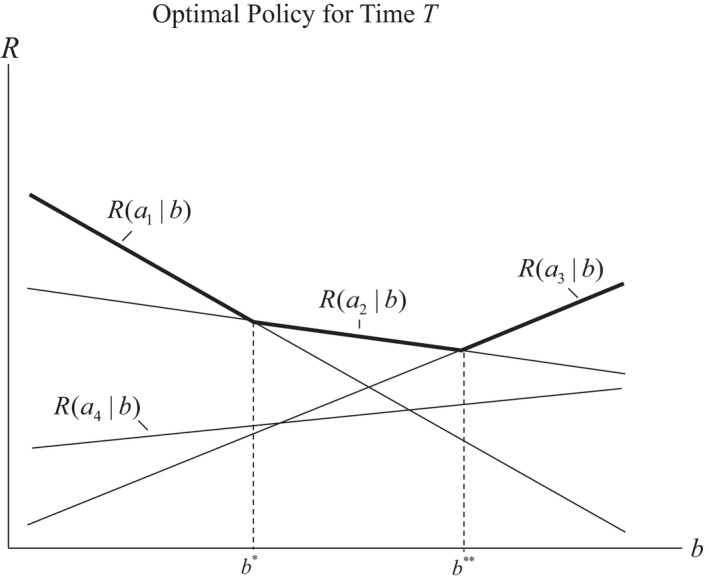
Value functions for terminal time *T*, with 2 states, 4 actions, and belief state $\left(b,1-b\right)$. Each action generates a different return function $R\left(a|b\right)={\mathrm{bR}}_{1}\left(a\right)+\left(1-b\right)R_{2}\left(a\right)$. Partitioning of belief space into 3 segments and the optimal actions for each are determined by which return function produces the largest value at each belief state. Optimal value function is indicated by darkened line segments.

A standard approach with POMDPs takes advantage of the fact that only finitely many policy trees are needed at any given time to define an optimal policy across the belief space (Smallwood & Sondik, 1973). Each tree defines a linear function, and optimization over the linear functions partitions the belief space into a finite number of segments such that optimal values are produced with the same linear function for all belief states in a given segment. One consequence is an optimal value function that is piecewise linear over the belief space (see, e.g., Figure 6). The vectors defining the piecewise linearity are called α – vectors, and those for a particular time *t* are denoted in aggregate by $L_{t}$.

By working inductively from the terminal time, it is possible to derive α – vectors (and their partitioning of belief space) at each time *t*, on the basis of previously identified α – vectors. The procedure for doing so begins at the terminal time *T*, where the optimal terminal value for belief state b is the maximum of $\sum_{x}b\left(x\right)R\left(a|x\right)$ for the possible actions *a*. The α – vectors for terminal time consist of the return vectors $R\left(a\right)$ with components $R\left(a|x\right),\;x\in X$ that produce a maximum average return for at least one belief state:

$$\begin{array}{c} V_{T}\left[b\right]=\sum_{x}b\left(x\right)\alpha \left(x\right)\;\\ =\max_{a\in A}\sum_{x}b\left(x\right)R\left(a|x\right). \end{array}$$

Maximization leads to a partition of the belief space into segments, such that the same action (and vector of returns) is optimal for all belief states in a segment (Figure 6). The set of all α – vectors for terminal time *T* is denoted by $L_{T}$.

Building on $L_{T}$, an inductive argument for time t≤T−1 utilizes previously identified α – vectors for stage t+1 to construct the α – vectors for *t*. With the form in Equation (8), $V_{t}\left[b\right]$ can be written as

$$V_{t}\left[b\right]=\sum_{x}b\left(x\right)\left\{R\left(a^{*}|x\right)+\lambda \sum_{o^{\prime }}P\left(o^{\prime }|x,a^{*}\right)\;\max_{\alpha \in L_{t+1}}\sum_{x^{\prime }}b_{a^{*},o^{\prime }}\left(x^{\prime }\right)\alpha \left(x^{\prime }\right)\right\},$$

which allows one to identify for a belief state *b* the α – vector at time *t* with components

$$R\left(a^{*}|x\right)+\lambda \sum_{o^{\prime }}P\left(o^{\prime }|x,a^{*}\right)\;\max_{\alpha \in L_{t+1}}\sum_{x^{\prime }}b_{a^{*},o^{\prime }}\left(x^{\prime }\right)\alpha \left(x^{\prime }\right).$$

Operationally, the inductive task is to find α – vectors in $L_{T}$ at the terminal time *T* as described above, then use $L_{T}$ to find the α – vectors in $L_{T-1}$ for time T−1, then use $L_{T-1}$ to find the α−vectors in $L_{T-2}$ for time T−2, and so on to the beginning of the timeframe.

Because an α – vector can be constructed as above for any belief state, the challenge at each time becomes one of selecting a limited number of belief states that will produce all the α – vectors needed to define $V_{t}\left[b\right]$ over the whole belief space. Most approaches to exact solutions for POMDPs are distinguished by the method of finding a set of belief states that will produce all the α – vectors. Two general approaches (Cassandra, 1994) are:

- at each time generate a superset $L_{t}^{+}$ of vectors that includes the set $L_{t}$ of α – vectors, then reduce $L_{t}^{+}$ to $L_{t}$ (e.g., Cassandra et al., 1997; Monahan, 1982; Zhang & Liu, 1997); and
- at each time create subsets $L_{t}^{-}$ of vectors that approximate the optimal value function, then grow the sets while eliminating dominated vectors to get $L_{t}$ (e.g., Cheng, 1988; Kaelbling et al., 1998).

In large part, methods for finding exact POMDP solutions do not scale well, and are tractable only for fairly small problems over a limited time (Littman, 2009). Fortunately, some ecological problems can be framed in ways that make them amenable to exact solutions. For larger problems, approximation methods that limit the search for optimal valuation are required (see Discussion).

## 6 INFINITE TIME HORIZONS

In this section, we extend the time horizon to allow for decision making over an unlimited amount of time. This is an important consideration because many problems are framed in terms of decision making that can sustain ecological systems indefinitely. Here we describe policy valuation that at any given time is based on expected values that accumulate over infinitely many future time steps. We show how policy and value differ between observable and partially observable MDPs with infinite time horizons.

The development thus far has been based on a time horizon $\left\{0,1,\ldots ,T\right\}$ with a known and finite terminal time *T*. Because conservation is so often framed in terms of sustaining ecological systems into the indefinite future, it is useful to consider management that continues over infinitely many decision periods, and identify steady‐state management policies that sustain resources indefinitely. In our waterfowl harvest example, we may wish to consider harvest strategies over an indefinitely long time horizon. With full observability and time discounting, the value function $V\left(x_{0}\right)=E\left[R\left(a_{0}|x_{0}\right)+\sum_{t=1}^{\infty }\lambda^{t}R\left(a_{t}|x_{t}\right)\right]$ has finite values, so optimal policies and values can be identified. Under partial observability optimal valuation can be approximated, and possibly determined exactly, depending on the structure of the harvest problem.

### 6.1 Infinite time horizon for observable MDPs

Optimal valuation for an observable process with infinitely many time steps can be obtained with a stationary policy consisting of state‐specific actions that are invariant to the time at which they are taken (Howard, 1960; Puterman, 1994). Let $\pi =\left[a\left(x\right)\right]$ represent such a policy, where the same action $a\left(x\right)$ is taken for state *x* irrespective of the time of its occurrence.

A process with stationary policy π can be represented in matrix form by a return vector $R_{\pi }=\left[r\left(a\left(x\right)\right)\right]$ and a matrix $P_{\pi }=\left[P(x^{\prime }|x,a\left(x\right))\right]$ of action‐specific transition probabilities. Optimal valuation is given in matrix form by

(10) $$V_{\pi^{*}}=\max_{\pi }{\left(I-\lambda P_{\pi }\right)}^{-1}R_{\pi },$$

with a corresponding optimal policy

$$\pi^{*}=\arg\;\underset{\pi }{\max}{\left(I-\lambda P_{\pi }\right)}^{-1}R_{\pi }$$

(see Appendix S1). A straightforward procedure for identifying optimal values and policies starts with the selection of an arbitrary policy π to approximate $\pi^{*}$, followed by the determination of values $V_{\pi }\left(x\right)$ by

$$V_{\pi }={\left(I-\lambda P_{\pi }\right)}^{-1}R_{\pi }.$$

The values $V_{\pi }$ then are used to identify a new policy $\pi^{\prime }$ by

$$\pi^{\prime }=\arg\;\underset{\pi }{\max}\left\{R_{\pi }+\lambda P_{\pi }V_{\pi }\right\},$$

and the new policy is used in turn to determine new values

$$V_{\pi^{\prime }}={\left(I-\lambda P_{\pi^{\prime }}\right)}^{-1}R_{\pi^{\prime }}.$$

Under mild conditions, recursive policy approximation and value determination can be shown to converge to $\pi^{*}$ and $V_{\pi^{*}}$, irrespective of the initial policy choice (Howard, 1960; Ross, 1970).

### 6.2 Infinite time horizon for partially observable MDPs

Value iteration for POMDPs, in which the α – vectors for one time are used to find α – vectors for the immediately preceding time, can be used to approximate, and sometimes identify, optimal policies and value functions for infinite time horizons (Poupart, 2005). Repeated value iteration produces values (and policies) that begin to converge, as increasingly discounted values for later rewards add less and less to the accumulated value. That is, the longer the duration of the system process, the smaller the difference between successive valuations, and the closer the value function gets to a stationary value function and policy (Cassandra, 1994).

In some but not all cases, the optimal value function for infinitely many time steps can be determined exactly in a limited number of steps, and described as a piecewise convex function with a limited set of α – vectors (Hansen, 1998; Sondik, 1978). In other cases, value iteration converges to the infinite horizon optimal value function only in the limit as the number of time steps increases without bound. For this situation the optimal value function will be convex in *b*, but not necessarily piecewise linear (Kaelbling et al., 1998; White & Harrington, 1980). In the latter case, repeated value iteration provides an approximation of the optimal infinite horizon value function, but the approximation can be arbitrarily close with enough iterations (Sawaki & Ichiwaka, 1978; Sondik, 1978).

## 7 MIXED OBSERVABILITY

In this section, we describe mixed observability models for situations in which only some state variables are observable. This is especially important in ecology because ecological systems often include both observable and unobservable attributes, and both can be important in ecological assessment and management. Here we develop adaptive management in the context of mixed observability, and further extend adaptive decision making to include nonstationarity over time.

It may be that some state variables in a system are observable and some are not. For example, the management of a nature preserve might involve conserving a threatened species that is not observable, and managing its wetland habitats that are. It is useful to account for such a mixture of observability conditions in designing management strategies.

Thus, consider a framework for a POMDP in which the system is characterized by two states $\left(x,y\right)$ with process transition probabilities $P\left(x^{\prime },y^{\prime }|x,y,a\right)$ and observations $o=\left(o_{x},o_{y}\right)$ with observation probabilities $f\left(o_{x}^{\prime },o_{y}^{\prime }|x^{\prime },y^{\prime },a\right)$. Assuming *x* and *y* are discrete with dimensions $n_{1}$ and $n_{2}$, one can treat this problem as a classical POMDP of dimension $n=n_{1}\times n_{2}$. The process probabilities

$$P\left(x^{\prime },y^{\prime },o_{x}^{\prime },o_{y}^{\prime }|x,y,a\right)=P\left(x^{\prime },y^{\prime }|x,y,a\right)f\left(o_{x}^{\prime },o_{y}^{\prime }|x^{\prime },y^{\prime },a\right)$$

can be used for valuation as described above.

This framework can be used to define a mixed observability MDP or MOMDP (Araya‐Lopez et al., 2010; Ong et al., 2010), in which the system state is separated into observable states *x* and unobservable states *y*. The observation probabilities for known states are given by

$$f\left(o_{x}^{\prime }|x^{\prime },y^{\prime },a,o_{y}^{\prime }\right)=\left\{\begin{array}{c} 1\;\text{if}\;o_{x}^{\prime }=x^{\prime }\;\\ 0\;\text{otherwise},\; \end{array}\right.$$

that is, observation $o_{x}^{\prime }$ coincides with the state $x^{\prime }$. On the other hand, observation $o_{y}^{\prime }$ is stochastically related to the unobservable state $y^{\prime }$ by

$$f\left(o_{y}^{\prime }|x^{\prime },y^{\prime },a,o_{x}^{\prime }\right)=f\left(o_{y}^{\prime }|x^{\prime },y^{\prime },a,x\right).$$

Assuming observations for *y* are not influenced by *x*, the transition probabilities are

$$P\left(x^{\prime },y^{\prime },o_{y}^{\prime }|x,y,a\right)=P\left(x^{\prime },y^{\prime }|x,y,a\right)f\left(o_{y}^{\prime }|y^{\prime },a\right)$$

with belief updates

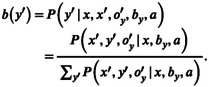




In the absence of an unobservable state *y*, a MOMDP problem is seen to reduce to an observable MDP, for which the system state *x* is observed (Figure 2). Alternatively, in the absence of an observable state *x* the problem reduces to a POMDP in state *y*, with an observation function $f\left(o_{y}^{\prime }|y^{\prime },a\right)$ (Figure 3). An important effect of factorization into observable and unobservable components is to reduce the dimensionality of the belief state space, which in turn reduces the computation time for finding solutions with POMDP solvers (Nicol et al., 2015).

### 7.1 MOMDPs and adaptive management

The MOMDP framework can be applied to adaptive management problems, which involve structurally uncertain systems and the reduction of structural uncertainty about system processes through management actions. Adaptive management is commonly described in terms of observable MDPs for which there is uncertainty about the transition structure or its parameters (Walters, 1986; Williams, 2009). For example, system dynamics may be characterized by one of several models, with uncertainty as to which is the most appropriate. Alternatively, there may be an accepted model but uncertainty about one of more model parameters, such as a population model with uncertain survival or reproduction rates. In either case, state transitions can be characterized with transition probabilities $P\left(x^{\prime }|x,a,y\right)$, where *y* denotes a particular model (or parameter value) and process uncertainty is expressed in terms a belief state $b_{y}$ over a discrete space of models or parameters (Williams, 2011).

This situation can be treated as a special case of a MOMDP, in which *x* represents the observable system state and *y* represents the unknown model or parameter value. When the process model is only partially observable and the system state is known, the decision process is sometimes called a hidden model MDP or hmMDP (Chadès et al., 2014; Pozzi et al., 2017).

In many adaptive management applications, the true process is held to be stationary over time and included in the model or parameter set. Monitoring of system status over time is assumed to reveal the actual state *x* at each monitoring event, with no other observations to inform $b_{y}$ besides the sequential monitoring of system status. In this situation, valuation becomes

(11) $$V_{\pi_{t}}\left(x,b_{y}\right)=R\left(a|x,b_{y}\right)+\sum_{x^{\prime }}P\left(x^{\prime }|x,a,b_{y}\right)V_{\pi_{t+1}}\left(x^{\prime },b_{y}^{\prime }\right)$$

with optimal valuation

$$V_{t}\left[x,b_{y}\right]=\max_{a}\left\{R\left(a|x,b_{y}\right)+\sum_{x^{\prime }}P\left(x^{\prime }|x,a,b_{y}\right)V_{t+1}\left[x^{\prime },b_{y}^{\prime }\right]\right\},$$

where

$$R\left(a|x,b_{y}\right)=\sum_{y}b\left(y\right)R\left(a|x,y\right),$$

$$P\left(x^{\prime }|x,a,b_{y}\right)=\sum_{y}b\left(y\right)P\left(x^{\prime }|x,a,y\right)$$

and

$$b_{y}^{\prime }\propto \sum_{y}P\left(x^{\prime }|x,a,y\right)b\left(y\right)$$

(Williams, 2011). Like POMDPs in general, this problem is PSPACE‐complete over finite horizons (Chadès et al., 2014), and thus is difficult to solve for any but small problems.

### 7.2 Nonstationary models

A useful generalization of hidden model MDPs allows for nonstationarity in the model structure, such that the true model (or parameter) is itself subject to change through time. For example, climate change can produce such nonstationarity, as climate trends alter system dynamics over time. Pollution, habitat fragmentation, disturbances and other factors can similarly affect ecological processes and lead to nonstationary dynamics.

Nonstationarity can be incorporated by allowing for the model structure to change through time as environmental and other factors change. One approach is to model the structural change (Nicol et al., 2015), by characterizing a change from a model (or parameter) *y* to $y^{\prime }$ by transition probabilities $P\left(y^{\prime }|y\right)$ and including the probabilities as an added source of change along with the state dynamics. An intuitive expression that includes both sources of change consists of the probabilities

$$P\left(x^{\prime },y^{\prime }|x,a,y\right)=P\left(y^{\prime }|y\right)P_{y^{\prime }}\left(x^{\prime }|x,a\right),$$

where state transitions from *x* to $x^{\prime }$ are based on model $y^{\prime }$ once a model change occurs with probability $P\left(y^{\prime }|y\right)$. Because there are two sources of structural uncertainty in this expression, namely model uncertainty for the prior and posterior models, it is necessary to account for both in valuation:

$$V_{\pi_{t}}\left(x|y,y^{\prime }\right)=R_{y}\left(a|x\right)+\sum_{x^{\prime }}P_{y^{\prime }}\left(x^{\prime }|x,a\right)V_{\pi_{t+1}}\left(x^{\prime }|y^{\prime }\right).$$

Letting $P\left(y^{\prime }|y\right)=b_{y}\left(y^{\prime }\right)$ and $b_{y}=\left[b\left(y^{\prime }|y\right)\right]$, the average value over the models $y^{\prime }$ is

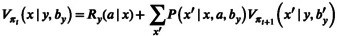

where

$$b_{y}^{\prime }\left(y^{\prime }\right)=\frac{b_{y}\left(y^{\prime }\right)P_{y^{\prime }}\left(x^{\prime }|x,a\right)}{P\left(x^{\prime }|x,a,b_{y}\right)}$$

with $P\left(x^{\prime }|x,a,b_{y}\right)=\sum_{y^{\prime }}b_{y}\left(y^{\prime }\right)P_{y^{\prime }}\left(x^{\prime }|x,a\right)$ and $b_{y}^{\prime }=\left[b_{y}^{\prime }\left(y^{\prime }\right)\right]$ (see Appendix S1). A second averaging over the models *y* produces

(12) $$V_{\pi_{t}}\left(x|b,b_{y}\right)=R\left(a|b\right)+\sum_{x^{\prime }}P\left(x^{\prime }|x,a,b_{y},b\right)V_{\pi_{t+1}}\left(x^{\prime }|b^{\prime },b_{y}^{\prime }\right)$$

where

$$b^{\prime }\left(y\right)=\frac{b\left(y\right)P\left(x^{\prime }|x,a,b_{y}\right)}{P\left(x^{\prime }|x,a,b_{y},b\right)}$$

with $P\left(x^{\prime }|x,a,b_{y},b\right)=\sum_{y}b\left(y\right)P\left(x^{\prime }|x,a,b_{y}\right)$ and $b^{\prime }=\left[b^{\prime }\left(y\right)\right]$ (see Appendix S1).

Equation (12) can be seen as a generalization of Equation (11) for valuation under stationarity; if $P\left(y^{\prime }|y\right)=b_{y}\left(y^{\prime }\right)$ is eliminated, Equation (12) reduces to valuation under stationarity as in Equation (11).

Mixed observability models offer opportunities to account for multiple uncertainty factors in ecological assessment and management, especially under current conditions of rapid environmental change due to climate change and other factors. In particular, there is real potential for advances in learning‐based adaptive management under nonstationary conditions. Additional features for consideration include the incorporation of partially observable states as well as system models (Fackler & Pacifici, 2014), and autocorrelations in trajectories of model structure over time (Memarzadeh et al., 2019).

## 8 CONTINUOUS STATES

In this section, we address the complexity added in POMDPs with a continuous state space. Although much of the modeling and analysis of POMDPs is based on an assumption that state variables range over discrete values, many ecological problems focus on states such as density rate and size, which can vary over a continuous range of values. Such a situation presents serious difficulties in formulating and evaluating policies under partial observability. We describe approaches for policy valuation under these conditions.

The restriction to discrete and finite states and observations clearly limits the range of ecological applications for POMDPs, since many ecological problems involve continuous state variables for which the solution methods for discrete decision processes are not applicable (Zhou et al., 2010). For example, our waterfowl harvest problem may be described in terms of continuous rather than discrete population status, where the population is modeled as a continuous Markov process with transitions from states over a continuous range to other states in that range. A different approach must be used to assess such a problem.

A key issue in the propagation and updating of a continuous belief state is that posterior belief states typically do not have the same functional form as the prior belief states. A possible solution is to approximate a continuous‐state POMDP with one over a discretized state space, and use the optimal policy for the resulting discrete‐state POMDP as a proxy for the continuous process (Hauskrecht, 2000; Zhou & Hansen, 2001). Other approaches involve gradient ascent (Meuleau et al., 1999; Ng & Jordan, 2000), neural networks (Bertsekas & Tsitsiklis, 1996; Sallans, 2000), and Monte Carlo simulation (Brooks & Williams, 2010; Thrun, 1999).

A promising new approach for handling continuous‐state POMDPs is “density projection,” so named because it involves the projection of belief states onto a set of parametrically defined probability distributions. With density projection, the belief states share a common functional form, and thus can be characterized by their parameters rather than by the probability masses for individual system states. Though Bayesian updating produces a posterior belief state that differs in form from its prior, the posterior is approximated with a proxy that is close to it and in the same family as the prior belief state.

The practical challenge of finding the best approximation for a posterior belief is achieved in density projection by identifying distribution parameters of the proxy that minimize the Kullback–Leibler divergence between the true and proxy distributions (Zhou et al., 2010). Zhou et al. (2010) show that for distributions in the exponential family, minimization of Kullback–Leibler divergence is obtained by matching the sufficient statistics of the true and approximate distributions. With the additional step of discretizing the parameter space and using a nearest‐neighbor approach to represent transitions between discrete parameter values, one can use solution approaches for discrete‐state POMDPs to find approximate solutions to the continuous‐time MDP (see Appendix S1).

By allowing continuous belief states to be characterized by probability density function parameters taking only a limited number of values, density projection goes a long way toward addressing the curse of dimensionality and expands dramatically the range of POMDP applications. The approach has been used to address structural uncertainty (Springborn & Sanchirico, 2013) as well as partial observability, where it was first applied informally to wildlife management by Moore (2008). Since then, there have been a number of biological examples (see Table 1 for examples).

## 9 EXAMPLES

In this section, we use simple examples involving control of a nuisance species to show how POMDPs build upon the framework and calculations for observable MDPs and produce piece‐wise linear optimal valuations.

### 9.1 Observable MDP example

To illustrate assessment of an observable MDP, consider a simple problem of controlling the abundance of a nuisance animal species, involving two states ($x_{1}$ for low abundance, $x_{2}$ for high abundance); three potential actions (no investment in conservation ($a_{1}$), temporary habitat alteration ($a_{2}$), and trapping and removal of animals ($a_{3}$)); and a model describing the consequences of these actions on the population status. The transition probabilities for each action are

$$P\left(a_{1}\right)=\left[P\left(x^{\prime }|x,a_{1}\right)\right]=\left[\begin{array}{cc} .3 & .7\\ 0 & 1 \end{array}\right]\;\left[\begin{array}{cc} \mathrm{low}\Rightarrow \mathrm{low} & \mathrm{low}\Rightarrow \text{high}\\ \text{high}\Rightarrow \mathrm{low} & \text{high}\Rightarrow \text{high} \end{array}\right]$$

$$P\left(a_{2}\right)=\left[P\left(x^{\prime }|x,a_{2}\right)\right]=\left[\begin{array}{cc} .8 & .2\\ .3 & .7 \end{array}\right]$$

$$P\left(a_{3}\right)=\left[P\left(x^{\prime }|x,a_{3}\right)\right]=\left[\begin{array}{cc} .6 & .4\\ .8 & .2 \end{array}\right]$$

Some patterns are noteworthy. In the absence of any conservation action, there is a high probability of transition from low to high abundance, but no chance of transition from high to low abundance. Habitat alteration produces smaller probabilities of transition from high to low abundance than trapping. And there are substantial probabilities that high abundance will remain unchanged even when a conservation action is undertaken.

Returns for this problem include immediate costs and benefits of conservation actions, as well as social perceptions about the appropriateness of an action. It is assumed that the cost of trapping is greater than that of temporary habitat alteration, that positive values accrue to both the reduction of abundance and the retention of low abundance, and that social perceptions and values vary with costs, success, and the type of action taken. The average return when action *a* is taken in state *x* is shown in Table 2.

**TABLE 2:** Immediate return for conservation action *a* given state *x*

		Action		
		$a_{1}$ (preserve)	$a_{2}$ (alter habitat)	$a_{3}$ (trap and remove)
State	$x_{1}$ (low)	$R\left(a_{1}|x_{1}\right)=14.5$	$R\left(a_{2}|x_{1}\right)=12.0$	$R\left(a_{3}|x_{1}\right)=10.0$
	$x_{2}$ (high)	$R\left(a_{1}|x_{2}\right)=5.0$	$R\left(a_{2}|x_{2}\right)=7.5$	$R\left(a_{3}|x_{2}\right)=5.5$

It is easy to see that at terminal time *T* the optimal value for a low population is $V_{T}\left[x_{1}\right]=\max_{a}R\left(a|x_{1}\right)=14.5$ with optimal action $a^{*}=a_{1}$. For a large population the optimal value is $V_{T}\left[x_{2}\right]=\max_{a}R\left(a|x_{2}\right)=7.5$ with optimal action $a^{*}=a_{2}$.

At time *T*–1 optimal valuation with discount factor λ=0.9 is given by

$$V_{T-1}\left[x\right]=\max_{a}\left\{R\left(a|x\right)+0.9\sum_{x^{\prime }}P\left(x^{\prime }|x,a\right)\;V_{T}\left[x^{\prime }\right]\right\},$$

with optimal value.

$$V_{T-1}\left[x_{1}\right]=\max\left\{23.1,23.8,22.5\right\}=23.8\;\text{for}\;a^{*}=a_{2}$$

for state $x_{1}$ and

$$V_{T-1}\left[x_{2}\right]=\max\left\{11.8,16.1,17.3\right\}=17.3\;\text{for}\;a^{*}=a_{3}$$

for state $x_{2}$. At time *T–*2 optimal valuation is given by

$$V_{T-2}\left[x\right]=\max_{a}\left\{R\left(a|x\right)+0.9\sum_{x^{\prime }}P\left(x^{\prime }|x,a\right)\;V_{T-1}\left[x^{\prime }\right]\right\},$$

with

$$V_{T-2}\left[x_{1}\right]=\max\left\{31.8,32.2,29.1\right\}=32.2\;\text{for}\;a^{*}=a_{2}$$

and

$V_{T-2}\left[x_{2}\right]=\max\left\{18.3,24.8,25.8\right\}=25.8\;\text{for}\;a^{*}=a_{3}$

A summary of the optimal strategy and valuation for three time steps is shown in Table 3.

**TABLE 3:** Optimal time‐specific values and conservation actions for state *x*

	Time		
	*T*–2	*T*–1	*T*
State $x_{1}$ (low)	$V_{t}\left[x_{1}\right]=32.2$; $a^{*}=a_{2}$	$V_{t}\left[x_{1}\right]=23.8$; $a^{*}=a_{2}$	$V_{t}\left[x_{1}\right]=14.5$; $a^{*}=a_{1}$
State $x_{2}$ (high)	$V_{t}\left[x_{2}\right]=25.8$; $a^{*}=a_{3}$	$V_{t}\left[x_{2}\right]=17.3$; $a^{*}=a_{3}$	$V_{t}\left[x_{2}\right]=7.5$; $a^{*}=a_{2}$

Backward recursion beyond *T*–2 generates a stationary policy $\pi =\left[a_{2},a_{3}\right]$ with habitat conservation $\left(a_{2}\right)$ for a small population and removal $\left(a_{3}\right)$ for a large population. These actions attempt to maintain the size of a small population and reduce the size of a large population over indefinitely many time steps. From Equation (10), the state‐specific optimal values for an infinite time horizon are $V\left[x_{1}\right]=126.5$ and $V\left[x_{2}\right]=130.1$.

### 9.2 Partially observable MDP example

An observable MDP can be extended to create a POMDP by allowing for partial observability with an observation function. For example, three possible observations, $o_{1},o_{2},\text{and}\;o_{3}$ (for, e.g., observed population counts that are low, medium, or high) might be associated with state‐specific probabilities (Table 4):

**TABLE 4:** Probabilities corresponding to observation *a* for a given state *x*

	Observation		
	$o_{1}$ (low)	$o_{2}$ (medium)	$o_{3}$ (high)
State $x_{1}$	$f\left(o_{1}|x_{1}\right)=0.1$	$f\left(o_{2}|x_{1}\right)=0.6$	$f\left(o_{3}|x_{1}\right)=0.3$
State $x_{2}$	$f\left(o_{1}|x_{2}\right)=0.5$	$f\left(o_{2}|x_{2}\right)=0.4$	$f\left(o_{3}|x_{2}\right)=0.1$

The observation probabilities combine with Markov transitions between states to define the POMDP transitions $P\left(o^{\prime },x^{\prime }|x,a\right)=f\left(o^{\prime }|x^{\prime }\right)\;P\left(x^{\prime }|x,a\right)$. With only two states, the belief state at any time can be described by a vector with a scalar value *b* for state $x_{1}$ and (1–*b*) for state $x_{2}$.

To illustrate optimal decision making with a POMDP, we again consider two states but allow a fourth action, for example, a combination of habitat alteration and removal. At terminal time *T*, there are no future values to consider, so the optimal value function for a given belief state is the maximum of the linear functions

$$V_{T}\left(a|b\right)=\mathrm{bR}\left(a|x_{1}\right)+\left(1-b\right)R\left(a|x_{2}\right)$$

where action *a* can be $a_{1},a_{2},a_{3}$ or $a_{4}$. Figure 6 displays four lines corresponding to value functions for the actions over the belief space [0,1]. Optimization over the actions partitions the belief space [0,1] into three segments that are defined by the intersections of three of the four lines (the function $V_{T}\left(a_{4}|b\right)$ is dominated over [0,1], and thus is not needed to describe the optimal value function). The figure makes clear that optimization produces a convex optimal value function $V_{T}\left[b\right]$ that is piecewise linear in *b*. Thus, $V_{T}\left[b\right]$ is given by $V_{T}\left(a_{1}|b\right)$ for belief states less than $b^{*}$; by $V_{T}\left(a_{3}|b\right)$ for belief states greater than $b^{**}$; and by $V_{T}\left(a_{2}|b\right)$ for belief states between $b^{*}$ and $b^{**}$.

The return vectors for the three value functions defining the optimal value function constitute the α – vectors for time *T*, with an α – vector corresponding to each of the three partition segments. With more actions the number of intersections tends to increase, so the number of segments in the partition of [0,1] and the number of α – vectors does as well. Countering this tendency is the fact that more dominated lines typically occur, which tends to reduce the count of α – vectors.

At time *T*–1, the optimal value $V_{T-1}\left[b\right]$ is produced with the algorithm for Equation (7) in the following steps:

1. for each action $a_{T-1}$ and combination $\left(o^{\prime },a_{T}\right)$, transform the return vector with components $R\left(a_{T}|x\right)$ into a vector with component $V^{\prime }\left(a_{T}|x,a_{T-1},o^{\prime }\right)=\sum_{x^{\prime }}P\left(o^{\prime },x^{\prime }|x,a_{T-1}\right)R\left(a_{T}|x^{\prime }\right)$;
2. maximize $V^{\prime }\left(a_{T}|b,a_{T-1},o^{\prime }\right)=\sum_{x}b\left(x\right)V^{\prime }\left(a_{T}|x,a_{T-1},o^{\prime }\right)$ over the actions $a_{T}$;
3. accumulate the results of step 2 over all observations $o^{\prime }$ and add the immediate return $R\left(a_{T-1}|b\right)$; and
4. maximize the result of step 3 over the actions $a_{T-1}$ to get $V_{T-1}\left[b\right]=\max_{a_{T-1}}\left\{R\left(a_{T-1}|b\right)+\lambda \sum_{o^{\prime }}\max_{a_{T}}V^{\prime }\left(a_{T}|b,a_{T-1},o^{\prime }\right)\right\}$.

Though the arithmetic in these steps can be tedious, the computations are actually simple. Because the functions $V^{\prime }\left(a_{T}|b,a_{T-1},o^{\prime }\right)$ are simply lines in two dimensions, the solution of the optimization simplifies to a piecewise linear value function in two dimensions.

**FIGURE 7:**
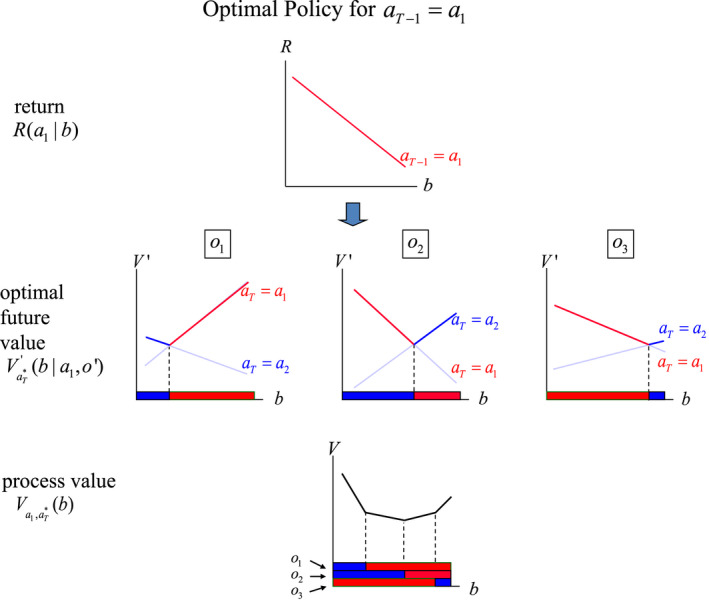
Valuation at time *T*–1 for a policy tree with root action $a_{1}$ and optimal sub‐policies thereafter. Graphs display (i) immediate returns $R\left(a_{1}|b\right)$; (ii) backcast values $V^{\prime }\left(a_{T}|b,a_{1},o^{\prime }\right)$ for each observation, along with partition segment cutpoints; and (iii) the accumulation of immediate returns and optimal backcast values over observations to get $V_{a_{1},\pi_{T}^{*}}\left(b\right)$.

**FIGURE 8:**
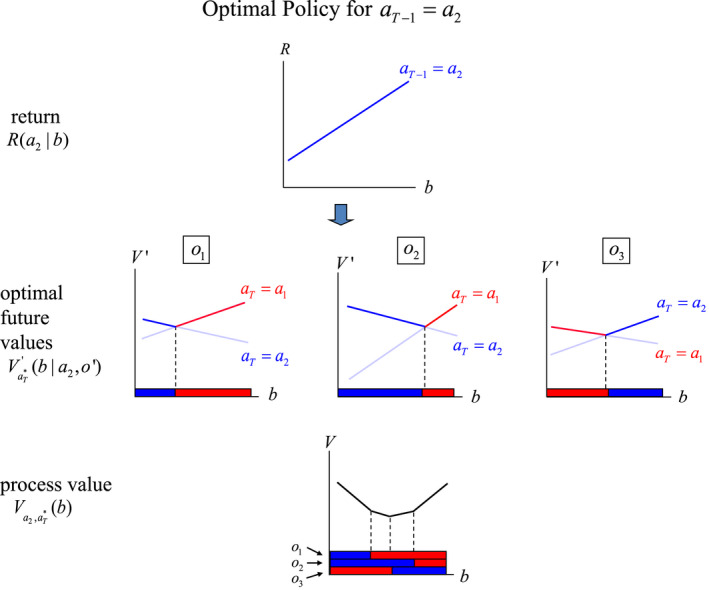
Valuation at time *T*–1 for a policy tree with root action $a_{2}$ and optimal sub‐policies thereafter. Graphs display (i) immediate returns $R\left(a_{2}|b\right)$; (ii) backcast values $V^{\prime }\left(a_{T}|b,a_{2},o^{\prime }\right)$ for each observation, along with partition segment cutpoints; and (iii) the accumulation of immediate returns and optimal backcast values over observations to get $V_{a_{2},\pi_{T}^{*}}\left(b\right)$.

For illustrative purposes consider only two actions $a_{1}$ and $a_{2}$, with immediate and average returns shown in Table 5 (also see Figures 7 and 8).

**TABLE 5:** Immediate returns for two actions, given two states. $R\left(a|b\right)$ corresponds to returns averaged over belief state *b*

	State		
	$x=x_{1}$	$x=x_{2}$	$R\left(a|b\right)$
Action $a_{1}$	$R\left(a_{1}|x_{1}\right)=2.3$	$R\left(a_{1}|x_{2}\right)=7.9$	$R\left(a_{1}|b\right)=7.9-5.6b$
Action $a_{2}$	$R\left(a_{2}|x_{1}\right)=8.1$	$R\left(a_{2}|x_{2}\right)=2.5$	$R\left(a_{2}|b\right)=2.5+5.6b$

For each action $a_{T-1}$ and observation $o^{\prime }$, the returns can be transformed with the probabilities $P\left(x^{\prime },o^{\prime }|x,a_{T-1}\right)$ as indicated in the Appendix S1, to produce linear functions $V_{a_{T}}^{\prime }\left(b|a_{T-1},o^{\prime }\right)$ shown in Table 6.

**TABLE 6:** Values $V_{a_{T}}^{\prime }\left(b|a_{T-1},o^{\prime }\right)$ at time *T*–1 for actions $\left(a_{T-1},a_{T}\right)$ and observation $o^{\prime }$ following $a_{T-1}$

		Observation		
		$o^{\prime }=o_{1}$	$o^{\prime }=o_{1}$	$o^{\prime }=o_{1}$
Action $a_{T-1}=a_{1}$	$a_{T}=a_{1}$	2.8+5.2b	7.9−6.8b	7.5−3.4b
	$a_{T}=a_{2}$	4.8−3.1b	0.2+6.4b	2.5+2.1b
Action $a_{T-1}=a_{2}$	$a_{T}=a_{1}$	4.3+3.8b	0.3+7.7b	6.2−2.4b
	$a_{T}=a_{2}$	6.2−2.3b	7.9−2.4b	3.9+2.1b

**FIGURE 9:**
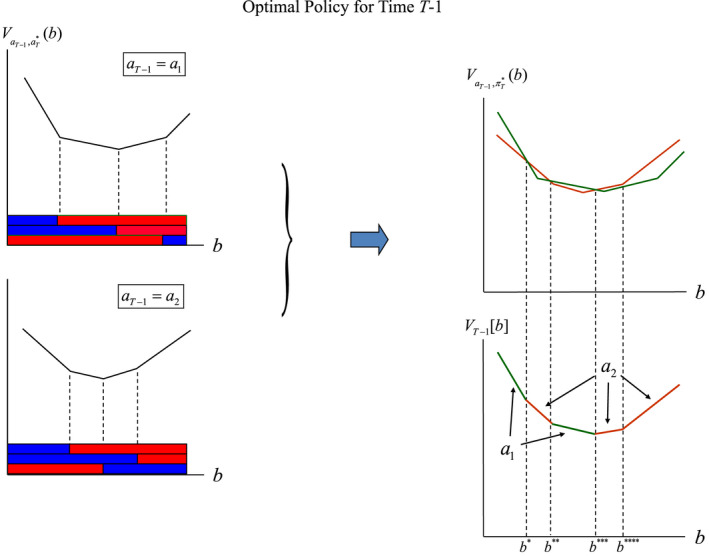
Combining the value functions $V_{a_{1},\pi_{T}^{*}}\left(b\right)$ and $V_{a_{2},\pi_{T}^{*}}\left(b\right)$ to produce optimal valuation $V_{T-1}\left[b\right]$ for time *T*–1. Partitioning of belief space is determined by the time *T* partitions for $V_{a_{1},\pi_{T}^{*}}\left(b\right)$ and $V_{a_{2},\pi_{T}^{*}}\left(b\right)$, and the intersection points of the 2 functions. The optimal action for belief states in each partition segment is determined by which of the 2 value functions produces the larger value.

Conditional on action $a_{T-1}$ and each observation $o^{\prime }$, optimal values for time *T* are then obtained by optimizing $V_{a_{T}}^{\prime }\left(b|a_{T-1},o^{\prime }\right)$ over $a_{T}$ (Figures 6, 7 and 7, 8), and a subsequent optimization over the actions at *T*–1 identifies the optimal value function and final partition of belief space (Figure 9).

The optimal partition of belief space [0,1] shown in Figure 9 includes several segments, with the same optimal policy for all belief states in a segment. The number of segments defined by the optimizations can be expected to increase with the number of potential actions.

For time *t* prior to *T*–1, the optimal value function for a general time identifies the maximum accumulated returns over the remaining time horizon for each belief state *b* starting at time *t*. Thus, the value function

$$\begin{array}{c} V_{t}\left[b\right]=\max_{a_{t}}\left\{R\left(a_{t}|b\right)+\lambda \sum_{o^{\prime }}P(o^{\prime }|b,a_{t})\;V_{\pi_{t+1|a_{t},o}^{*}}\left(b_{a_{t},o^{\prime }}\right)\right\}\\ =\max_{a_{t}}\left\{R\left(a_{t}|b\right)+\lambda \sum_{o^{\prime }}P(o^{\prime }|b,a_{t})\;V\left[b_{a_{t},o^{\prime }}\right]\right\} \end{array}$$

is optimized by a two‐step procedure to get $V_{t+1}\left[b\right]=\max_{\pi_{t+1}}V_{\pi_{t+1}}\left(b\right)$ at time *t* + 1, followed by a second optimization over the actions $a_{t}$. The solution gives an optimal action $a_{t}^{*}$ and associated optimal value $V_{t}\left[b\right]$ for each belief state *b* for each time.

The identification of optimal values and policies in the foregoing invasive species problem is greatly simplified by the small number of population sizes, actions and observations. However, even with this simplification the number of segments defined by the optimizations can become exponentially large as the duration of the process is extended.

## 10 DISCUSSION

We have focused on partially observable Markov decision processes in the context of managing and monitoring ecological systems, when there is only limited understanding of ecological status. Markov transitions are usually assumed to occur in a discrete state space, with controls that influence both rewards and transitions among states. All aspects of the control problem must be adapted to partial observability, including state transitions, valuation, and the tracking of system status by means of belief states. These features add considerable complexity, in large part because of the expansion of a discrete state space under complete observability into a continuous state belief space under partial observability.

A technical treatment of partial observability with POMDPs is rarely undertaken in ecology and ecological assessments, despite the almost universal presence of uncertainty about a system's status. In fact, a POMDP framework is applicable across a broad spectrum of ecological problems involving populations, communities, ecosystems, and habitats. It also can be applied naturally to decision making about monitoring protocols and programs, by including actions in the observation function $f\left(o^{\prime }|x^{\prime },a\right)$ that allow a manager to address whether, when and how to conduct monitoring so as to maximize conservation value.

Several factors contribute to the limited use of POMDPs in ecology and ecological management. Challenges include the complexity of the POMDP framework and the notation needed to characterize it; difficulties in interpreting solutions for all but very simple problems; the inability to scale up exact methods to problems with large numbers of states and lengthy time horizons; and importantly, the lack of explanatory documentation and examples that can help potential users (Chadès et al., 2021).

All combinations of finitely many states, actions, and observations can be listed for any belief state in a POMDP. However, it is not possible to do so for all the infinitely many belief states in the continuous belief space of a POMDP, and thus it is not possible to enumerate values over the continuous space. Most approaches for solving POMDPs utilize the piecewise linear structure of the optimal value function, which allows the partitioning of belief space into segments and the use of a single linear function to produce optimal values for all belief states in a given segment. The challenge is then to identify the partition segments and associated linear functions for each time step.

Numerous solution methods have been formulated for POMDPS, each with its own advantages and limitations. Several approaches, such as the witness algorithm (Kaelbling et al., 1998; Littman, 1996) and incremental pruning (Cassandra et al., 1997; Zhang & Liu, 1997), produce exact solutions, but scale poorly and generally can be used for only a limited class of small problems. Ad hoc procedures (e.g., use of observation moments as if they are actual system states, gridding of belief space and valuation at grid points to approximate $V\left[b\right]$) are relatively straightforward, but may perform poorly even for small problems (Cassandra, 1994). Point‐based value iteration (Pineau et al., 2006; Spaan & Vlassis, 2005), a popular approach that approximates the value function with a limited number of systematically identified belief states, has become increasingly available via recent web applications (Pascal et al., 2020). Outstanding issues are the range and density of the belief states that are included, and convergence rates and costs of the approach with increasing scale.

There are some key assumptions underlying POMDPs that limit their use. One is that transitions among states are Markovian, which restricts the usefulness of POMDPs to ecological systems not exhibiting hysteresis and other lags in resource processes and valuations. Another is that the sets X,A, and *O* of process states, actions, and observations are assumed to be finite. One approach for problems with continuous actions and observations is to discretize their range of values (Nicol & Chadès, 2012), but the solutions produced may be sensitive to the discretization rules. Another uses density projection to approximate solutions, as described earlier.

Additional assumptions are that the structure of the ecological system is fixed and fully known. Structural uncertainty can be accommodated in a POMDP framework as discussed in Section 7.1 (Memarzadeh & Boettiger, 2018; Williams, 2009, 2011), which allows for adaptive learning as management is pursued (Fackler et al., 2014; Peron et al., 2017). Structural nonstationarity can also be modeled in terms of mixed observability, as suggested in Section 7.2. Artificial intelligence shows promise for nonstationary decision processes (Nicol et al., 2015).

For problems that meet the basic assumptions, POMDPs add realism in framing the management of ecological systems, by recognizing that they are almost never observed in their entirety and that sampling produces only stochastic estimators of ecological status (Williams & Brown, 2019). Though relatively few in number, applications of POMDPs in ecology have grown in recent years, as resource analysts and managers increasingly seek to account for uncertainty. Applications are aided by ongoing developments in theory, solution techniques, and computing capacity (e.g., Dujardin et al., 2017), as well as improvements in the display of policy graphs (Ferrer‐Mestres et al., 2020, 2021). In particular, finding efficient approaches to approximate optimal solutions for large problems is a rapidly growing area of research. Coupled with advances in the fast‐evolving field of ecological sampling and estimation, POMDPs hold considerable promise for more effective ecological management.

## AUTHOR CONTRIBUTIONS

**Byron Williams:** Conceptualization (lead); methodology (lead); writing – original draft (lead); writing – review and editing (lead). **Eleanor Brown:** Conceptualization (supporting); funding acquisition (lead); writing – review and editing (supporting).

## CONFLICT OF INTEREST

The authors declare no conflict of interest.

## Supporting information


Appendix S1

## Data Availability

No data were used in the preparation of this manuscript. Therefore, no data are available for access.
