# Supplementary material for: Partial observability and management of ecological systems
Source: Ecol Evol. 2022 Sep 13;12(9):e9197. doi: 10.1002/ece3.9197 (PMC9468910; doi:10.1002/ece3.9197)
Supplement: Supplementary file 1 — Appendix S1 [file ECE3-12-e9197-s001.docx]

**APPENDIX**

**VALUES AND POLICIES: OBSERVABLE MDP**

**Value**

Value for an observable MDP can be expressed with policy tree $\pi_{t}=\left\{a_{t},\pi_{t+1|a_{t},x'}\right\}$as

$$\begin{matrix} V_{\pi_{t}}(x)=E\left[\sum_{\tau=t}^{T} \lambda^{\tau-t}R\left(a_{\tau}|x_{\tau}\right)|x\right] \\ =R\left(a_{t}|x\right)+\lambda\sum_{x'} P(x'|x,a_{t})E\left[\sum_{\tau=t+1}^{T} \lambda^{\tau-t}R\left(a_{\tau}|x_{\tau}\right)|x'\right] \\ =R\left(a_{t}|x\right)+\lambda\sum_{x'} P(x'|x,a_{t})V_{\pi_{t+1|a_{t},x'}}(x'). \end{matrix}$$

**Optimal value**

Optimal valuation is given by a 2-part optimization, by first optimizing posterior returns over policies for time *t*+1 and then optimizing the sum of prior and posterior returns over actions for time *t*:

$$\begin{matrix} V_{t}\left[x\right]=\max_{\pi_{t}} V_{\pi_{t}}\left(x\right) \\ =\max_{\pi_{t}} \left\{R\left(a_{t}|x\right)+\sum_{x'} P(x'|x,a_{t})V_{\pi_{t+1}|a_{t}}(x')\right\} \\ =\max_{a_{t}} \left\{R\left(a_{t}|x\right)+\lambda\sum_{x'} P(x'|x,a_{t}) V_{\pi_{t+1}^{*}}\left(x'\right)\right\} \\ =\max_{a_{t}} \left\{R\left(a_{t}|x\right)+\lambda\sum_{x'} P(x'|x,a_{t}) \max_{\pi_{t+1}} V_{\pi_{t+1}}\left(x'\right)\right\} \\ =\max_{a_{t}} \left\{R\left(a_{t}|x\right)+\lambda\sum_{x'} P(x'|x,a_{t}) V[x']\right\}.\text{ } \end{matrix}$$

**VALUES AND POLICIES: POMDP**

**Value**

Value for a POMDP with a particular observation$o'$can be expressed with policy tree$\pi_{t}=\left\{a_{t},\pi_{t+1|a_{t},o'}|o'\in O\right\}$as

$$\begin{matrix} V_{\pi_{t}}(x|o') =R(a_{t}|x)+\lambda\sum_{x'} P(x'|x,a_{t},o')V_{\pi_{t+1|a_{t},o'}}(x') \\ =R(a_{t}|x)+\lambda\sum_{x'} \frac{P(o',x'|x,a_{t})}{P(o'|x,a_{t})}V_{\pi_{t+1|a_{t},o'}}(x'), \end{matrix}$$

and the average of$V_{\pi_{t}}(x|o')$over all observations gives the value function

$$\begin{matrix} V_{\pi_{t}}(x) =R(a_{t}|x)+\lambda\sum_{o'} P(o'|x,a_{t})\sum_{x'} \frac{P(o',x'|x,a_{t})}{P(o'|x,a_{t})}V_{\pi_{t+1|a_{t},o'}}(x') \\ =R(a_{t}|x)+\lambda\sum_{o'} \sum_{x'} P(o',x'|x,a_{t})V_{\pi_{t+1|a_{t},o'}}(x') \\ =R(a_{t}|x)+\lambda\sum_{x'} P(x'|x,a_{t})\sum_{o'} f(o'|x',a_{t})V_{\pi_{t+1|a_{t},o'}}(x'). \end{matrix}$$

Then$V_{\pi_{t}}(x)$is averaged over the states *x* to give the value function for belief state *b* as

$$\begin{matrix} V_{\pi_{t}}(b) =\sum_{x} b(x)\left\{R(a_{t}|x)+\lambda\sum_{x'} P(x'|x,a_{t})\sum_{o'} f(o'|x',a_{t})V_{\pi_{t+1|a_{t},o'}}(x')\right\} \\ =R(a_{t}|b)+\lambda\sum_{o'} \sum_{x'} P(x',o'|b,a_{t})V_{\pi_{t+1|a_{t},o'}}(x'). \end{matrix}$$

Alternative expressions of$V_{\pi_{t}}(b)$can be obtained by:

- using transformed future values
- using updated belief states

$$\begin{matrix} V_{\pi_{t}}(b) =R(a_{t}|b)+\lambda\sum_{o'} \sum_{x'} P(o',x'|b,a_{t})V_{\pi_{t+1|a_{t},o'}}(x') \\ =R(a_{t}|b)+\lambda\sum_{o'} P(o'|b,a_{t})\sum_{x'} \frac{P(x',o'|b,a_{t})}{P(o'|b,a_{t})}V_{\pi_{t+1|a_{t},o'}}(x') \\ =R(a_{t}|b)+\lambda\sum_{o'} P(o'|b,a_{t})\sum_{x'} b_{a_{t},o'}(x')V_{\pi_{t+1|a_{t},o'}}(x') \\ =R(a_{t}|b)+\lambda\sum_{o'} P(o'|b,a_{t})V_{\pi_{t+1|a_{t},o'}}(b_{a_{t},o'}) \end{matrix}$$

These expressions for$V_{\pi_{t}}(b)$produce the same value for every belief state.

**Optimal value**

Optimal value for a POMDP can be obtained with either of the above approaches, as follows.

Using transformed values:

For a given belief state *b* at time *t*,

1. Transform$V_{\pi_{t+1|a_{t},o'}}(x')$into new values$V_{\pi_{t+1}}^{'}\left(x|a_{t},o'\right)=\sum_{x'} P(o',x'|x,a_{t})V_{\pi_{t+1|a_{t},o'}}(x')$.
2. Average the values from step 1 over *b* to get $V_{\pi_{t+1}}^{'}\left(b|a_{t},o'\right)=\sum_{x} b(x)V_{\pi_{t+1}}^{'}\left(x|a_{t},o'\right)$
3. Optimize$V_{\pi_{t+1}}^{'}\left(b|a_{t},o'\right)$over$\pi_{t+1}$to get$\max_{\pi_{t+1}} V_{\pi_{t+1}}^{'}\left(b|a_{t},o'\right)$
4. Discount the aggregate of optimal values from step 3 and add$R(a_{t}|b)$to get

$$Q_{t}\left(b|a_{t}\right)=R(a_{t}|b)+\lambda\sum_{o'} \max_{\pi_{t+1}} V_{\pi_{t+1}}^{'}\left(b|a_{t},o'\right)$$

1. Optimize results from step 4 over$a_{t}$to get

$$\begin{matrix} V_{t}[b]=\max_{a_{t}} Q_{t}\left(b|a_{t}\right) \\ =\max_{a_{t}} \left\{R(a_{t}|b)+\lambda\sum_{o'} \max_{\pi_{t+1}} V_{\pi_{t+1}}^{'}\left(b|a_{t},o'\right)\right\}. \end{matrix}$$

Using updated belief states:

For a given belief state *b* at time *t*,

1. Update *b* to get$b_{a_{t},o'}$, and average$V_{\pi_{t+1}}(x)$over$b_{a_{t},o'}$to get$V_{\pi_{t+1}}(b_{a_{t},o'})$
2. Optimize$V_{\pi_{t+1}}(b_{a_{t},o'})$over$\pi_{t+1}$to get$\max_{\pi_{t+1}} V_{\pi_{t+1}}(b_{a_{t},o'})$
3. Average the optimal values from step 2 over$P(o'|b,a_{t})$to get

$$\sum_{o'} P(o'|b,a_{t})\max_{\pi_{t+1}} V_{\pi_{t+1}}(b_{a_{t},o'})$$

1. Discount the average from step 3 and add$R(a_{t}|b)$to get $R(a_{t}|b)+\lambda\sum_{o'} P(o'|b,a_{t})\max_{\pi_{t+1}} V_{\pi_{t+1}}(b_{a_{t},o'})$
2. Optimize results from step 4 over$a_{t}$to get $V_{t}[b]=\max_{a_{t}} \left\{R(a_{t}|b)+\lambda\sum_{o'} P(o'|b,a_{t})\max_{\pi_{t+1}} V_{\pi_{t+1}}(b_{a_{t},o'})\right\}.$

These expressions for$V_{t}[b]$produce the same optimal value for every belief state.

**OPTIMAL VALUATION FOR AN INFINITE TIME HORIZON MDP**

Optimal values for an observable MDP with$T=\infty$can be obtained by optimizing over stationary policies$\pi=[a(x)]$consisting of time-invariant actions$a(x)$. An observable process with policy$\pi$is represented in matrix form by a return vector$R_{\pi}$and a matrix$P_{\pi}$of action-specific transition probabilities:

$$\begin{matrix} V_{\pi}=E\left[\sum_{t=0}^{\infty} \lambda^{t}R_{\pi}\right] \\ =R_{\pi}+\lambda P_{\pi}E\left[\sum_{t=0}^{\infty} \lambda^{t}R_{\pi}\right] \\ =R_{\pi}+\lambda P_{\pi}V_{\pi}, \end{matrix}$$

or

$$V_{\pi}=\left(I-\lambda P_{\pi}\right)^{-1}R_{\pi}.$$

Optimal valuation is then obtained by

$V_{\pi^{*}}=\max_{\pi} \left(I-\lambda P_{\pi}\right)^{-1}R_{\pi}$,

with the corresponding optimal policy

$\pi^{*}=\arg\max_{\pi} \left(I-\lambda P_{\pi}\right)^{-1}R_{\pi}$.

**STANDARD VS EXTENDED MARKOV DECISION MODELS**

Consider 2 transition models, distinguished by the timing of observations and their role in state transitions.

**Standard model**

Observations are tied to posterior states and used to update beliefs, without directly affecting the state transition probabilities.

Transition sequence

Observations occur after state transitions,

$$x,a\to x'\to o'$$

Transition probability

Transition probabilities$P(x'|x,a)$are not influenced observations$o'$, which are tied to posterior state$x'$though$f(o'|x',a)$:

$P(x',o'|x,a)=f(o'|x',a)P(x'|x,a)$.

Belief update

$$\begin{matrix} b_{a,o'}(x')=P(x'|b,a,o') \\ =\frac{P(x',o'|b,a)}{P(o'|b,a)} \end{matrix}$$

Value function

$$\begin{matrix} V_{\pi_{t}}(b)=\sum_{x} b(x)V_{\pi_{t}}(x) \\ =\sum_{x} b(x)\left[R(a|x)+\lambda\sum_{x'} \sum_{o'} P(x',o'|x,a) V_{\pi_{t+1|a,o'}}(x')\right] \\ =R(a|b)+\lambda\sum_{x'} \sum_{o'} P(x',o'|b,a) V_{\pi_{t+1|a,o'}}(x') \\ =R(a|b)+\lambda\sum_{x'} \sum_{o'} P(o'|b,a)b_{a,o'}(x') V_{\pi_{t+1|a,o'}}(x') \\ =R(a|b)+\lambda\sum_{o'} P(o'|b,a) V_{\pi_{t+1|a,o'}}(b_{a,o'}) \end{matrix}$$

In words, the value function combines immediate and future value, averaged over observations$o'$.

**Extended model**

Observations are tied to the prior states, and serve an active role in affecting state transition probabilities.

Transition sequence

Observations occur before state transitions,

$$x,a\to o\to x'$$

Transition probability

Transition probabilities$P(x'|x,a,o)$are directly influenced observations$o$, which are tied to the prior state *x* though$f(o|x,a)$:

$$P(x',o|x,a)=f(o|x,a)P(x'|x,a,o)$$

Belief update

$$\begin{matrix} b_{a,o}(x')=P(x'|b,a,o) \\ =\frac{P(x',o|b,a)}{f(o|b,a)} \end{matrix}$$

Value function

$$\begin{matrix} V_{\pi_{t}}(b)=\sum_{x} b(x)V_{\pi_{t}}(x) \\ =\sum_{x} b(x)\left[R(a|x)+\lambda\sum_{x'} \sum_{o} P(x',o|x,a) V_{\pi_{t+1|a,o}}(x')\right] \\ =R(a|x)+\lambda\sum_{x'} \sum_{o} P(x',o|b,a) V_{\pi_{t+1|a,o}}(x') \\ =R(a|b)+\lambda\sum_{x'} \sum_{o} f(o|b,a)b_{a,o}(x') V_{\pi_{t+1|a,o}}(x') \\ =R(a|x)+\lambda\sum_{o} f(o|b,a) V_{\pi_{t+1|a,o}}(b_{a,o}) \end{matrix}$$

A comparison of value functions for the standard and extended models shows they differ only in the use of prior and posterior observations.

**VALUATION UNDER NON-STATIONARITY**

Assume non-stationary system dynamics that are governed by

$P(x',y'|x,a,y)=P(y'|y)P_{y'}(x'|x,a)$,

in which state transitions from *x* to$x'$are based on model$y'$once a model change occurs with probability$P(y'|y)$.

Process value for a specific pair of models *y* and$y'$is

$V_{\pi_{t}}\left(x|y,y'\right)=R_{y}(a|x)+\sum_{x'} P_{y'}(x'|x,a)V_{\pi_{t+1}}\left(x'|y'\right)$.

Letting$P(y'|y)=b_{y}(y')$and$b_{y}=\left[b(y'|y)\right]$, the average value over the models$y'$is

$$\begin{matrix} V_{\pi_{t}}\left(x|y,b_{y}\right)=R_{y}(a|x)+\sum_{y'} b_{y}(y')\sum_{x'} P_{y'}(x'|x,a)V_{\pi_{t+1}}\left(x'|y,y'\right) \\ =R_{y}(a|x)+\sum_{x'} \sum_{y'} b_{y}(y')P_{y'}(x'|x,a)V_{\pi_{t+1}}\left(x'|y,y'\right) \\ =R_{y}(a|x)+\sum_{x'} \sum_{y'} b_{y}'(y')P(x'|x,a,b_{y})V_{\pi_{t+1}}\left(x'|y,y'\right) \\ =R_{y}(a|x)+\sum_{x'} P(x'|x,a,b_{y})\sum_{y'} b_{y}'(y')V_{\pi_{t+1}}\left(x'|y,y'\right) \\ =R_{y}(a|x)+\sum_{x'} P(x'|x,a,b_{y})V_{\pi_{t+1}}\left(x'|y,b_{y}'\right) \end{matrix}$$

where

$$b_{y}'(y')=\frac{b_{y}(y')P_{y'}(x'|x,a)}{P(x'|x,a,b_{y})}$$

with$P(x'|x,a,b_{y})=\sum_{y'} b_{y}(y')P_{y'}(x'|x,a)$from Bayes’ theorem and $b_{y}'=\left[b_{y}^{'}(y')\right]$.

A second averaging over the models *y* produces

$$\begin{matrix} V_{\pi_{t}}\left(x|b,b_{y}\right)=\sum_{y} b(y)V_{\pi_{t}}\left(x|y,b_{y}\right) \\ =\sum_{y} b(y)\left[R_{y}(a|x)+\sum_{x'} P(x'|x,a,b_{y})V_{\pi_{t+1}}\left(x'|y,b_{y}'\right)\right] \\ =R(a|b)+\sum_{x'} \sum_{y} b(y)P(x'|x,a,b_{y})V_{\pi_{t+1}}\left(x'|y,b_{y}'\right) \\ =R(a|b)+\sum_{x'} \sum_{y} b'(y)P(x'|x,a,b_{y},b)V_{\pi_{t+1}}\left(x'|y,b_{y}'\right) \\ =R(a|b)+\sum_{x'} P(x'|x,a,b_{y},b)\sum_{y} b'(y)V_{\pi_{t+1}}\left(x'|y,b_{y}'\right) \\ =R(a|b)+\sum_{x'} P(x'|x,a,b_{y},b)V_{\pi_{t+1}}\left(x'|b',b_{y}'\right) \end{matrix}$$

where $b'(y)=\frac{b(y)P(x'|x,a,b_{y})}{P(x'|x,a,b_{y},b)}$

with$P(x'|x,a,b_{y},b)=\sum_{y} b(y)P(x'|x,a,b_{y})$and$b'=\left[b'(y)\right]$.

**DENSITY PROJECTION WITH CONTINUOUS STATES**

Density projection can be used with continuous-state POMDPs to project belief states onto a set of parametrically defined probability distributions. Belief states therefore share a common functional form, and characterized by distribution parameters. Though posterior updating produces a belief state that differs in form from its prior, the posterior belief state is approximated with a proxy that is close to it and in the same family as the prior belief state.

Finding the best approximation for a posterior belief is achieved in density projection by identifying distribution parameters of the proxy that minimize the Kullback-Leibler divergence between the true and proxy distributions. Minimization of Kullback-Leibler divergence is obtained by matching the sufficient statistics of the true and approximate distributions. Discretizing the parameter space and using a nearest-neighbor approach to represent transitions between discrete parameter values allows the use of solution approaches for discrete-state POMDPs to find approximate solutions to the continuous-time MDP.

A formal problem statement assumes a belief state *b* that is in an exponential family$\Omega$of distributions$f(x|\theta)$defined by parameter$\theta$, with parameter values restricted to$\theta_{1},...,\theta_{m}$. The single-step return for belief state$b=f(x|\theta_{i})$is$E[R(a|b)]=\tilde{R}(a,\theta_{i})$, and transitions between belief states *b* and$b'$ can be simulated with particle filtering (Arulampalam et al. 2002) and mapped to a member of the exponential family via density projection. The resulting projection distribution$\hat{P}(\theta|\theta_{i},a)$is then approximated by the distribution with the nearest neighbor parameter in$\left\{\theta_{1},...,\theta_{m}\right\}$, which produces transition probabilities$\tilde{P}(\theta_{j}|\theta_{i},a)$ that approximate$P(b'|b,a)$.
